# Adipocyte-expressed SIRT3 manipulates carnitine pool to orchestrate metabolic reprogramming and polarization of macrophages

**DOI:** 10.1038/s41419-025-07699-6

**Published:** 2025-05-15

**Authors:** Jiali Chen, Fei Zhou, Lei Zhang, Ruohan Lou, Cangman Zhang, Jianbo Wan, Xiaojun Ma, Ligen Lin

**Affiliations:** 1 State Key Laboratory of Quality Research in Chinese Medicine, Institute of Chinese Medical Sciences, University of Macau, Taipa, Macao; 2 Guangdong Provincial Key Laboratory of Medical Immunology and Molecular Diagnostics, Guangdong Medical University, Dongguan, China; 3 The Department of Endocrinology and Metabolism, The First Affiliated Hospital of Zhengzhou University, Zhengzhou, China; 4 Department of Pharmaceutical Sciences, Faculty of Health Sciences, University of Macau, Taipa, Macao

**Keywords:** Chronic inflammation, Metabolic disorders, Mechanisms of disease, Metabolic syndrome, Obesity

## Abstract

Obesity is accompanied with accumulation and pro-inflammatory polarization of macrophages in adipose tissue (AT), leading to systematical inflammation and insulin resistance. Impaired lipid metabolism and endocrine function in adipocytes is recognized as a culprit in the onset of adipose tissue inflammation. Lipid levels can be managed via inhibiting both synthesis and transport or via increasing fatty acid oxidation (FAO). The deacetylase Sirtuin 3 (SIRT3) participates in inflammatory responses via regulating mitochondrial function and FAO. Herein, an AT-specific SIRT3 overexpression mice model (AT-SIRT3OE) was generated using adeno-associated virus transduction. AT-specific SIRT3 overexpression did not alter body weight or adiposity in either regular chow diet or high-fat diet (HFD) fed mice. AT-SIRT3OE mice exhibited improved insulin sensitivity in HFD-fed mice, through alleviating infiltration of macrophage and pro-inflammatory macrophage polarization in the epididymal AT. The metabolomics analysis indicated that SIRT3 overexpressed adipocytes accumulated more L-carnitine (LC) and less long-chain acylarnitines in the medium. Furthermore, SIRT3 directly deacetylates and activates carnitine palmitoyltransferase 2 (CPT2), an obligate step in mitochondrial long-chain FAO, to enhance the LC turnover pool in adipocytes, which in turn promoted lipid metabolism and anti-inflammatory polarization in macrophages. Collectively, our study provided new evidence that adipocyte-expressed SIRT3 alleviates inflammatory crosstalk between adipocytes and macrophages through manipulating LC pool. Activating SIRT3 in adipocytes could be a potential strategy to alleviate obesity-related metabolic diseases.

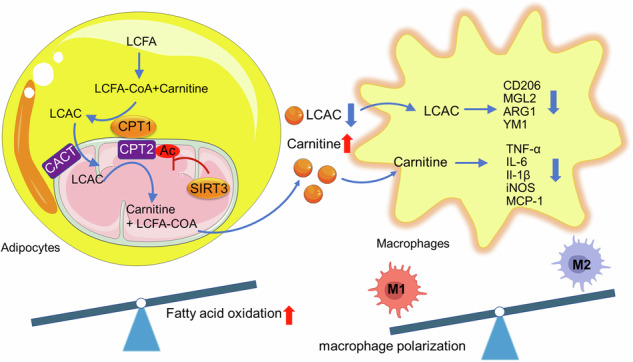

## Introduction

Long-term excessive energy causes the expansion of adipose tissue (AT), ultimately leading to overweight and obesity [1]. Obesity has been a public epidemic worldwide. In obese subjects, overpopulated immune cells (mainly macrophages) and dysfunctional adipocytes secrete a variety of cytokines and chemokines, causing low-grade long-term inflammation in AT, which plays a determining role in metabolic diseases, such as cardiovascular diseases, liver steatosis and type 2 diabetes [2]. The accumulation of macrophages and their pro-inflammatory polarization are hallmarks of the obesity-associated AT inflammation, in turn resulting in the development of systematic inflammation, dysfunctional insulin action and metabolic disorders [3].

Adipose tissue macrophages (ATMs) can be separated into the classically activated macrophages (pro-inflammatory macrophage) and the alternatively activated macrophages (anti-inflammatory macrophage) [4]. Plasticity is a hallmark of ATMs which enables them to react to surrounding microenvironment. Pro-inflammatory macrophages are increased in obese AT, while in lean AT, anti-inflammatory macrophages are the dominant one [5, 6]. Pro-inflammatory and anti-inflammatory macrophages exhibit particularly different metabolic characteristics: pro-inflammatory macrophages rely more on glycolysis for quick ATP generation while anti-inflammatory macrophages depend on oxidative phosphorylation (OXPHOS) [7, 8]. Similarly, ATMs in lean subjects are supported by fatty acid oxidation (FAO), glycolysis and glutaminolysis (anti-inflammatory macrophage), while ATMs in obese subjects depend more on glycolysis (pro-inflammatory macrophage) [9]. Cellular metabolism alters the microenvironment via generating and/or depleting metabolites, to regulate macrophage polarization [10–12]. Immune metabolites [13], such as serine [14], succinate [15], α-ketoglutarate [16], L-carnitine (LC) [17] and fatty acids (FAs) [18], are capable to manipulate polarization by remodeling intracellular metabolism in macrophages.

FA composition in AT influences both altered immune response and inflammatory signaling through different mechanisms [19]. Obese individuals often exhibit lower FAO rates, considered as a risk factor [20–22]. SIRT3, one of the seven nicotinamide adenine dinucleotide (NAD^+^)-dependent deacetylases in mammals, is mainly located within mitochondrial matrix, regulating differentiation, insulin sensitivity, inflammatory responses and lipid metabolism in adipocytes [23, 24]. SIRT3 expression is downregulated in obese patients, and deletion of SIRT3 leads to increased mitochondrial oxidative stress, reduced energy expenditure and worsened glucose metabolism, resulting in accelerated obesity under a high-fat diet (HFD) feeding [25, 26]. SIRT3 in β-cells regulates insulin secretion, and modulates hepatic lipid metabolism via the release of 5-hydroxytryptamine in HFD-fed mice [27]. Several lines of evidence indicated that SIRT3 deacetylates long-chain acyl-CoA dehydrogenase (LCAD), a critical enzyme for long-chain FA degradation, and carnitine palmitoyltransferase 2 (CPT2), a key enzyme for FA transfer, to enhance FAO [28–31]. Dysfunctional SIRT3 causes the accumulation of long-chain acylcarnitines (LCAC). Consistently, the levels of plasma LCAC are higher in patents of obesity and diabetes [32]. Accumulation of LCACs is harmful to mitochondria and can activate inflammation [33].

It is currently unclear how adipocyte-expressed SIRT3 manipulates macrophage polarization in AT of obese individuals. Therefore, we carried out untargeted metabolomics profiling in culture medium from SIRT3 overexpressed adipocytes and found higher level of LC and lower levels of acylcarnitines, which in turn orchestrate metabolic reprogramming and polarization in macrophages.

## Materials and methods

### Generation of adipocyte-specific SIRT3 overexpression mice

The environmental conditions in the animal facility were: temperature range of 21‒23 °C, humidity range of 40–50%, 12 h light/dark cycle, and free access to food and water. Adipocyte protein 2 (AP2) was chosen as the adipocyte-specific promotor [34]. The mouse SIRT3 (NM_001177804) was integrated into the GV585 vector (AP2 promotor-MCS-EGFP-3Flag-SV40 PolyA). Recombinant adeno-associated serotype 9 viruses with *Ap2* promoter for SIRT3 overexpression in adipocytes (AAV-*Ap2*-SIRT3) or the empty vector (AAV-*Ap2*) were acquired from GeneChem Co, Ltd (Shanghai, China). AAV-*Ap2*-SIRT3 or AAV-*Ap2* (5 × 10^10^ vg/mice) were administered via direct injection in the tail vein in mice at the first and the sixth week of the experiment, to generate adipose tissue-specific SIRT3 overexpression (AT-SIRT3OE) and control (AT-NC) mice, respectively.

### Experimental procedure of HFD-induced obese mice

8–10 week-old male C57BL/6J mice were purchased from the animal facility of the Faculty of Health Sciences, University of Macau. The mice were administrated with viral solution through intravenous tail vein injections. After a recovery period of 3 days, AT-NC and AT-SIRT3OE mice were randomly separated into two groups, each 6 mice, and fed with an HFD (calorie 4.5 kcal/g, Trophic Animal Feed High-Tech Co., Nantong, Jiangsu, China) and regular chow diet (RD, calorie 2.35 kcal/g), respectively, for consecutive 13 weeks [35]. Food intake and body weight were recorded every week. After 16 h fasting, blood was collected from tail vein, and sera were harvested after centrifuged at 3000 × *g* for 10 min at room temperature and stored at −80 °C. Epididymal white adipose tissues (eWAT), inguinal WAT (iWAT) and the liver were harvested. One parts of eWAT and the liver were fixed in 4% paraformaldehyde, and the rest of tissues was immediately frozen in liquid nitrogen and preserved at −80 °C.

### Cell culture

Bone marrow-derived macrophages (BMDMs) were harvested from the femur and tibia of C57BL/6J mice and cultured in Dulbecco’s modified Eagle’s medium (DMEM) containing 10% fetal bovine serum (FBS, Gibco, Carlsbad, CA, USA) and 10% L929 cell-conditioned medium, and maintained in a humidified incubator with 5% CO_2_ at 37 °C for 7 days [36]. Subsequently, the BMDMs were induced with interleukin-4 (IL-4, 40 ng/mL) or lipopolysaccharide (LPS, 100 ng/mL, Sigma-Aldrich, St. Louis, MO, USA) and interferon-γ (IFN-γ, 20 ng/mL) for 24 h to induce anti-inflammatory or pro-inflammatory polarization, respectively.

3T3-L1 preadipocytes were obtained from American Type Cell Collection (ATCC, Manassas, VA, USA), and maintained in DMEM supplemented with 10% calf serum (HyClone, Logan, UT, USA) and 1% penicillin–streptomycin (P/S, Gibco). 3T3-L1 preadipocytes were differentiated into mature adipocytes as described previously [37]. Briefly, 2 days post-confluent 3T3-L1 preadipocytes incubated with DMEM containing 10% FBS, 1 μM dexamethasone (Sigma-Aldrich), 0.5 mM 1-methyl-3-isobutylxanthine (Sigma-Aldrich) and 5 μg/mL insulin (Sigma-Aldrich). After 2 days, the medium was replaced to DMEM containing 10% FBS and 5 μg/mL insulin for 6 days. The culture medium was changed every 2 days.

### Generation of SIRT3 overexpressed and CPT2 knockdown cell lines

The pcDNA3.1-SIRT3 plasmid was generated as previously described [38]. Briefly, 3T3-L1 cells at 50% confluence were transfected with 10 μg plasmids (pcDNA3.1 or pcDNA3.1-SIRT3) using Lipofectamine 3000 reagent (Thermo Fisher Scientific, Grand Island, NY, USA). 24 h post transfection, 800 μg/mL G418 was added to select positive cells for 14 days. The medium was changed every other day. Thereafter, cells were pooled together for further experiments.

shRNAs targeting CPT2 were synthesized (CPT2KD-A: 5′-GCTGCCTATCCCTAAACTTGA-3′; CPT2KD-B: 5′-GCGGTTTCTGAAGACACTTCG-3′; CPT2KD-C: 5′-GGTTTGATAAGTCCTTTAACC-3′, CPT2KD-D: 5′-GCTGCAATGTCTCCTCCTACT-3′) and inserted into the pGPU6/GFP/Neo vector (Genepharma, Shanghai, China). 3T3-L1 cells at 50% confluence were transfected with 6 µg shRNA using Lipofectamine 3000 reagent for 18 h. Cells were incubated with fresh medium for 36 h and pooled together for further experiments.

### Macrophage-adipocyte co-culture

Macrophage-adipocyte co-culture was performed as described previously [39]. Mature 3T3-L1 adipocytes were washed with DMEM twice and then incubated in DMEM supplemented with 0.2% endotoxin and FA-free BSA for 48 h. Subsequently, the adipocyte conditioned medium (CM) was obtained by centrifugation of the medium at 4000 × *g* for 10 min. Then, BMDMs were exposed to adipocyte CM for 24 h, and harvested for further studies.

Macrophage migration assay was performed using 24-well transwell plates and inserts with an 8 μm membrane pore size (Millipore, Bedford, MA, USA). Mature 3T3-L1 adipocytes were maintained in the lower chamber and BMDMs (10 × 10^5^ cells/well) were cultured in the upper chamber. After co-cultured for 12 h at 37 °C, the macrophages on the lower surface of inserts were fixed with 4% formaldehyde for 30 min, stained with DAPI, and counted according to the previously established protocol [40].

### Flow cytometry

BMDMs were collected using 0.05% trypsin (Gibco) and resuspended in phosphate-buffered saline (PBS, Gibco). 1 × 10^6^ cells were incubated with F4/80-FITC, CD11c-PE and CD206-APC antibodies (BD Biosciences, Franklin Lakes, NJ USA) for 30 min on ice. After washing with PBS thrice, the stained cells were analyzed using BD-Cytoflex flow cytometer (BD Biosciences). The data of flow cytometry was analyzed using FlowJo software.

### Seahorse XF Mito fuel flex test

The dependency, capacity, and flexibility of cells to oxidize three major mitochondrial fuels (glucose, glutamine, and long chain FAs) were determined using a Seahorse XF Mito Fuel Flex Test Kit (103260-100, Agilent Technologies, Santa Clara, CA, USA) on a Seahorse Bioscience XF24-3 Extracellular Flux Analyzer (Agilent Technologies). The BMDMs and 3T3-L1 preadipocytes (5 × 10^4^ cells per well) were placed in XF24-well microplates (Agilent Technologies). 3T3-L1 preadipocytes were induced to full differentiation. The differentiated BMDMs were treated with LC and PC for 24 h, respectively. Then, the cells were switched to seahorse XF DMEM base medium and incubated in the absence of CO_2_ at 37 °C for 1 h. Subsequently, the cells were treated with BPTES (3 μM, a glutaminase inhibitor that blocks the glutamine oxidation pathway), etomoxir (4 μM, a CPT1A inhibitor, which is crucial for the transport of long-chain FAs from cytosol into mitochondria for β oxidation), or UK5099 (2 μM, an inhibitor of the glucose oxidation pathway that blocks the mitochondrial pyruvate carrier) successively, and oxygen consumption rate (OCR) was measured prior to and after the addition of each inhibitor. Data were recorded with XF Wave software. Fuel flexibility is calculated by subtracting the fuel dependency from the fuel capacity for the pathway of interest. Protein was quantified using a BCA Protein Assay Kit, and data were normalized to the total protein content.

### CPT2 activity

CPT2 activity in cell lysates was determined by using commercial CPT2 activity colorimetric quantitative detection kits (Shenzhen Ziker Biological Technology Co., Ltd., Shenzhen, China), according to the manufacturer’s instruction.

### Statistical analysis

Studies were designed to generate groups of equal size and no data points were excluded from the analysis in any experiment. The *n* value for each experiment was shown in the figure legend. Cells and mice were randomly assigned to each treatment group and experiment. All the quantifications and data analysis were blinded, and the analyst didn’t know the origin of the data during statistical analysis. The outliers were included in data analysis and presentation. Data normalization was undertaken to control for sources of variation of baseline parameters and to allow comparison of the magnitude of drug effects in different conditions. The units of a variable were determined as fold mean of the controls. Group size is the number of independent values, and data displaying a normal distribution was expressed as mean ± SEM based on at least three independent experiments and analyzed by GraphPad Prism 9.0 (GraphPad Software, San Diego, CA). The statistical significance of the differences between various treatments was measured by either the two tailed Student’s t test or one-way ANOVA with Tukey’s multiple comparisons test, considering *p* < 0.05 as a statistically significant difference.

## Results

### AT-specific overexpression of SIRT3 reverses HFD-induced insulin resistance independent of adiposity

**Fig. 1:**
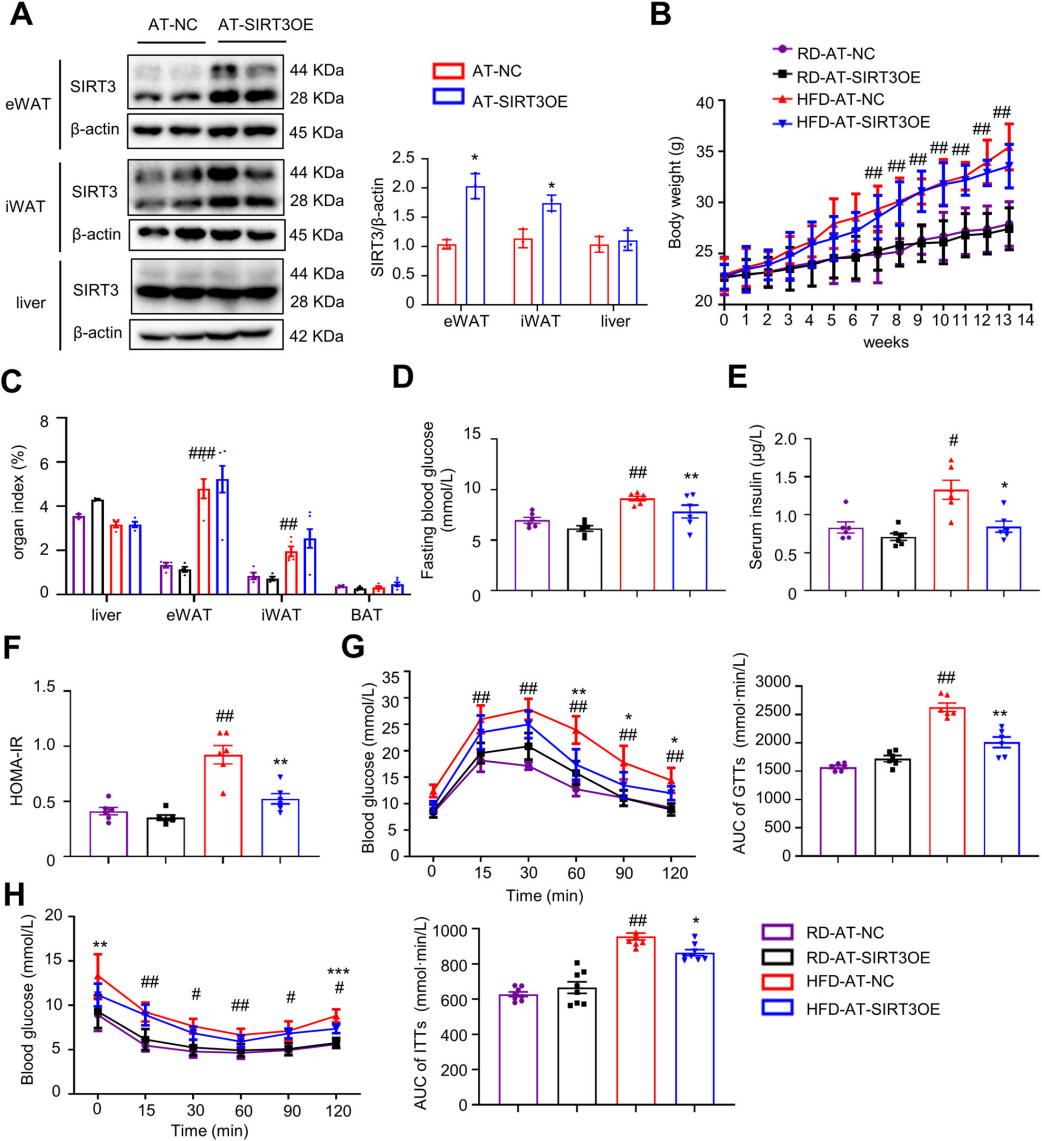
AT-SIRT3OE improves insulin sensitivity independent of adiposity in HFD-fed obese mice. **A** The protein expression of SIRT3 in eWAT, iWAT and the liver from AT-NC and AT-SIRT3OE mice. *n* = 4 independent experiments. **B** Body weight of mice with RD or HFD feeding. *n* = 6 mice per group. **C** Organ index of eWAT, iWAT, the liver and BAT (organ index = organ weight/body weight × 100%). *n* = 6 mice per group. **D** Fasting blood glucose level. *n* = 6 mice per group. **E** Serum insulin level after 16 h fasting. *n* = 6 mice per group. **F** The HOMA-IR index. *n* = 6 mice per group. **G** Glucose tolerance tests after 11 weeks of RD or HFD feeding and AUC (area under curve) of GTTs. *n* = 6 mice per group. **H** Insulin tolerance tests after 12 weeks of RD or HFD feeding and AUC of ITTs. *n* = 8 mice per group. Data are expressed as means ± SEM. ^#^*p* < 0.05, ^##^*p* < 0.01, ^###^*p* < 0.001, HFD-AT-NC vs. RD-AT-NC; *p < 0.05, **p < 0.01, AT-NC vs. AT-SIRT3OE.

To uncover the insulin-sensitizing effect of SIRT3 in AT, adipocyte-specific SIRT3 overexpressed mice were generated using an AAV delivered mouse SIRT3 under the control of the AP2 promoter. The Western blotting results demonstrated that the SIRT3 protein expression in eWAT and iWAT was greatly higher in AT-SIRT3OE mice, when compared with that of AT-NC mice, but not in the liver (Fig. 1A). The increased SIRT3 mRNA expression in eWAT was confirmed by qPCR results (Fig. S1A). HFD feeding caused more body weight increase than the RD feeding, while the body weight (Fig. 1B) and food intake (Fig. S1B) between AT-SIRT3OE and AT-NC mice were almost no change, in either RD or HFD feeding. Additionally, AT-SIRT3OE did not affect the mass of the liver, eWAT, iWAT or brown adipose tissue (BAT) (Fig. 1C). Next, the effect of AT specific SIRT3 overexpression on insulin sensitivity was evaluated. HFD-feeding significantly elevated the levels of fasting blood glucose and serum insulin compared with the RD-feeding; and AT-SIRT3OE obviously reversed the levels of blood glucose and serum insulin in HFD-fed mice, but not RD-fed mice (Fig. 1D, E). Calculation of the Homeostasis model assessment of basal insulin resistance (HOMA-IR) values suggested that AT-specific SIRT3 overexpression improved insulin sensitivity in HFD-feeding (Fig. 1F). During glucose tolerance tests (GTTs), the glucose disposal rate was interrupted in HFD-fed mice, and AT-SIRT3OE greatly accelerated the glucose clearance rate in HFD-fed mice, but not in RD-fed mice (Fig. 1G). Similarly, the results of insulin tolerance tests (ITTs) suggested that HFD feeding interrupted insulin action compared with the RD-fed mice, and AT-SIRT3OE enhanced insulin sensitivity in HFD-fed mice (Fig. 1H). In summary, AT-specific SIRT3 overexpression reverses HFD-induced insulin resistance independent of adiposity or energy intake.

### AT-specific SIRT3 overexpression alleviates the accumulation of macrophages and their pro-inflammatory polarization in eWAT from HFD-induced obese mice

**Fig. 2:**
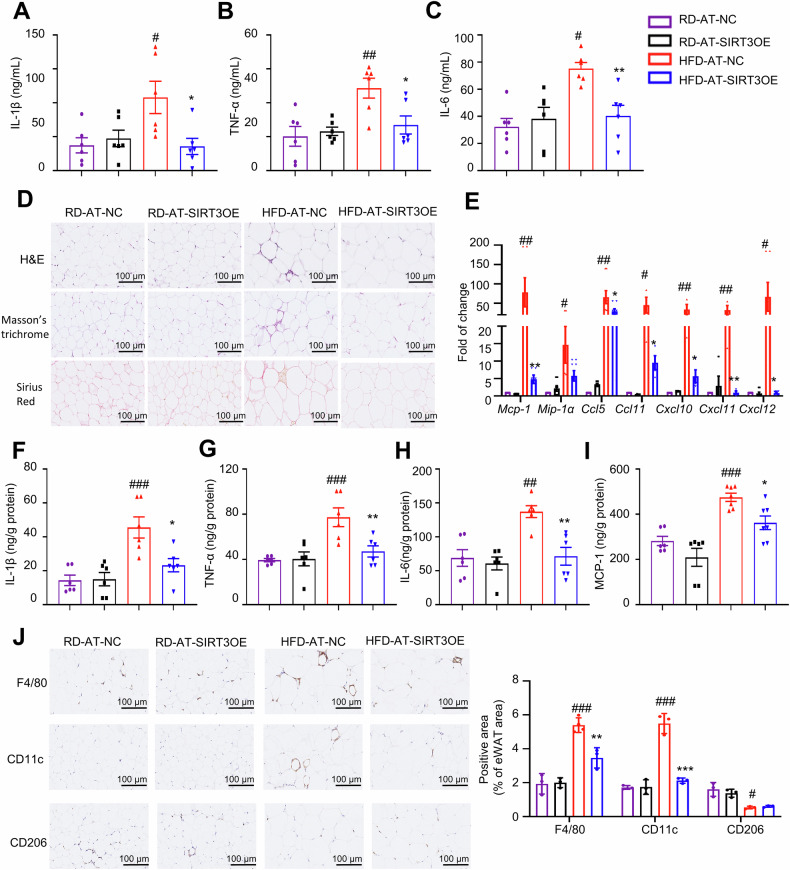
AT-SIRT3OE alleviates macrophage infiltration and pro-inflammatory macrophage polarization in the eWAT of obese mice. The serum levels of IL-1β (**A**) TNF-α (**B**) and IL-6 (**C**) were determined by ELISA kits. *n* = 6 mice per group. **D** Representative images of H&E staining, Masson’s trichrome staining (collagenous connective tissue fibers, blue-purple) and Sirius Red staining (collagen I/III fibers, pale pink) of eWAT. Original magnification, ×20 (top). *n* = 4 replicates. **E** The mRNA expression of *Mcp-1*, *Mip-1α*, *Ccl5*, *Ccl11*, *Cxcl10*, *Cxcl11* and *Cxcl12* in eWAT. *n* = 6 mice per group. The levels of IL-1β (**F**) TNF-α (**G**) IL-6 (**H**) and MCP-1 (**I**) in eWAT were determined by ELISA kits. *n* = 6 mice per group. (**J**) Immunohistochemical staining of F4/80, CD11c and CD206 in eWAT. Scale bar = 100 μm. The bar graphs represent the percentage of positive stained areas. *n* = 4 replicates. Data are expressed as means ± SEM. ^#^*P* < 0.05, ^##^*p* < 0.01, ^###^*p* < 0.001, HFD-AT-NC vs. RD-AT-NC; ^*^P < 0.05, ^**^P < 0.01, ^***^P < 0.001, HFD-AT-NC vs. HFD-AT-SIRT3OE.

In obese subjects, AT secretes various pro-inflammatory cytokines, causing systematic inflammation and insulin resistance [41]. In comparison of the RD-fed mice, the levels of interleukin (IL)-1β, tumor necrosis factor-α (TNF-α) and IL-6 in serum were obviously higher in HFD-fed mice, which were almost reversed in AT-SIRT3OE mice (Fig. 2A‒C). AT inflammation is characterized by the accumulation of macrophage and polarization towards the pro-inflammatory type [42]. We next investigated whether AT-specific SIRT3 overexpression mitigates AT inflammation. Compared with the RD-fed mice, more big adipocytes and increased number of infiltrated macrophages were found in eWAT from HFD-induced obese mice, based on the H&E staining results, which were obviously mitigated in eWAT from AT-SIRT3OE mice (Fig. 2D). In eWAT of HFD-fed mice, the collagen deposition and collagen fibers were increased, mainly formed in the fibrotic bundles around adipocytes, based on the Masson’s trichrome staining and Sirius Red staining results; as expected, the abnormal deposition of collagen was almost abolished in eWAT from AT-SIRT3OE mice (Fig. 2D). Macrophage inflammatory protein-1α (MIP-1α) and monocyte chemoattractant protein-1 (MCP-1) play the essential roles for AT macrophage recruitment [43]. The mRNA expression of chemokines in eWAT from obese mice, including *Mcp-1, Mip-1α*, C-C motif chemokine (*Ccl*) 5, *Ccl11*, C-X-C motif chemokine ligand 10 (*Cxcl10*), *Cxcl11* and *Cxcl12*, was significantly upregulated, compared with those of normal lean mice, and AT specific SIRT3 overexpression markedly reversed the expression (Fig. 2E), suggesting that SIRT3 overexpression suppressed macrophage chemotaxis. Furthermore, the ELISA results showed that the increased levels of IL-1β, TNF-α, IL-6 and MCP-1 in eWAT from obese mice were obviously abrogated in eWAT from AT-SIRT3OE mice (Fig. 2F‒2I). Compared with the normal lean mice, the numbers of infiltrating macrophages and pro-inflammation-polarized macrophages were obviously increased in eWAT from HFD-induced obese mice, evidenced by the immunohistofluorescent staining of F4/80 (macrophage marker) and CD11c (pro-inflammatory macrophage marker), which were mostly abolished in AT-SIRT3OE mice (Fig. 2J). On the contrary, the level of CD206 (anti-inflammatory macrophage marker) was significantly dampened in AT from HFD-fed mice, and AT-specific SIRT3 overexpression did not alter obviously (Fig. 2J). Collectively, AT specific SIRT3 overexpression mitigates AT inflammation by reducing macrophage infiltration and suppressing macrophage polarization towards pro-inflammatory type in eWAT from obese mice.

### SIRT3 overexpression mitigates adipocytes CM-induced inflammatory responses in macrophages

**Fig. 3:**
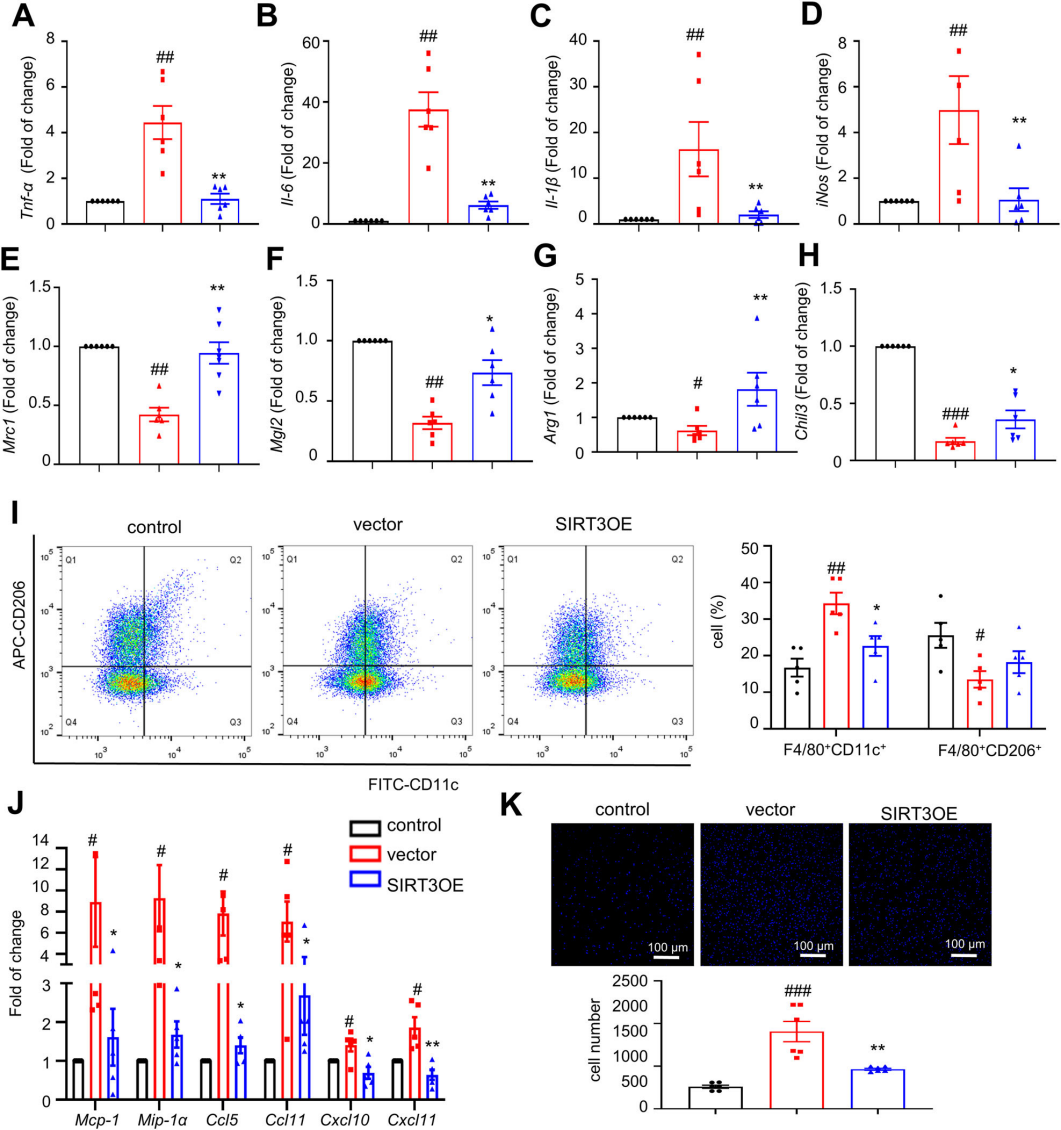
SIRT3 overexpression attenuates adipocytes CM induced inflammatory responses in macrophages. The mRNA expression of *Tnf-α* (**A**) *Il-6* (**B**), *Il-1β* (**C**) and *iNos* (**D**) in BMDMs. The mRNA expression of *Mrc1* (**E**) *Mgl2* (**F**) *Arg1* (**G**) and *Chil3* (**H**) in BMDMs. *n* = 6 independent experiments. **I** The F4/80/CD11c double-positive and F4/80/CD206 double-positive macrophages were analyzed by flow cytometry. *n* = 5 independent experiments. **J** The mRNA expression of *Mcp-1*, *Mip-1α*, *Ccl5*, *Ccl11*, *Cxcl10* and *Cxcl11* in BMDMs. *n* = 6 independent experiments. **K** Transwell migration of macrophages towards CM from vector and SIRT3OE adipocytes. *n* = 6 independent experiments. Data are expressed as means ± SEM. ^#^*P* < 0.05, ^##^*p* < 0.01, ^###^*p* < 0.001, vector vs. control; *P < 0.05, **P < 0.01, vector vs. SIRT3OE.

**Fig. 4:**
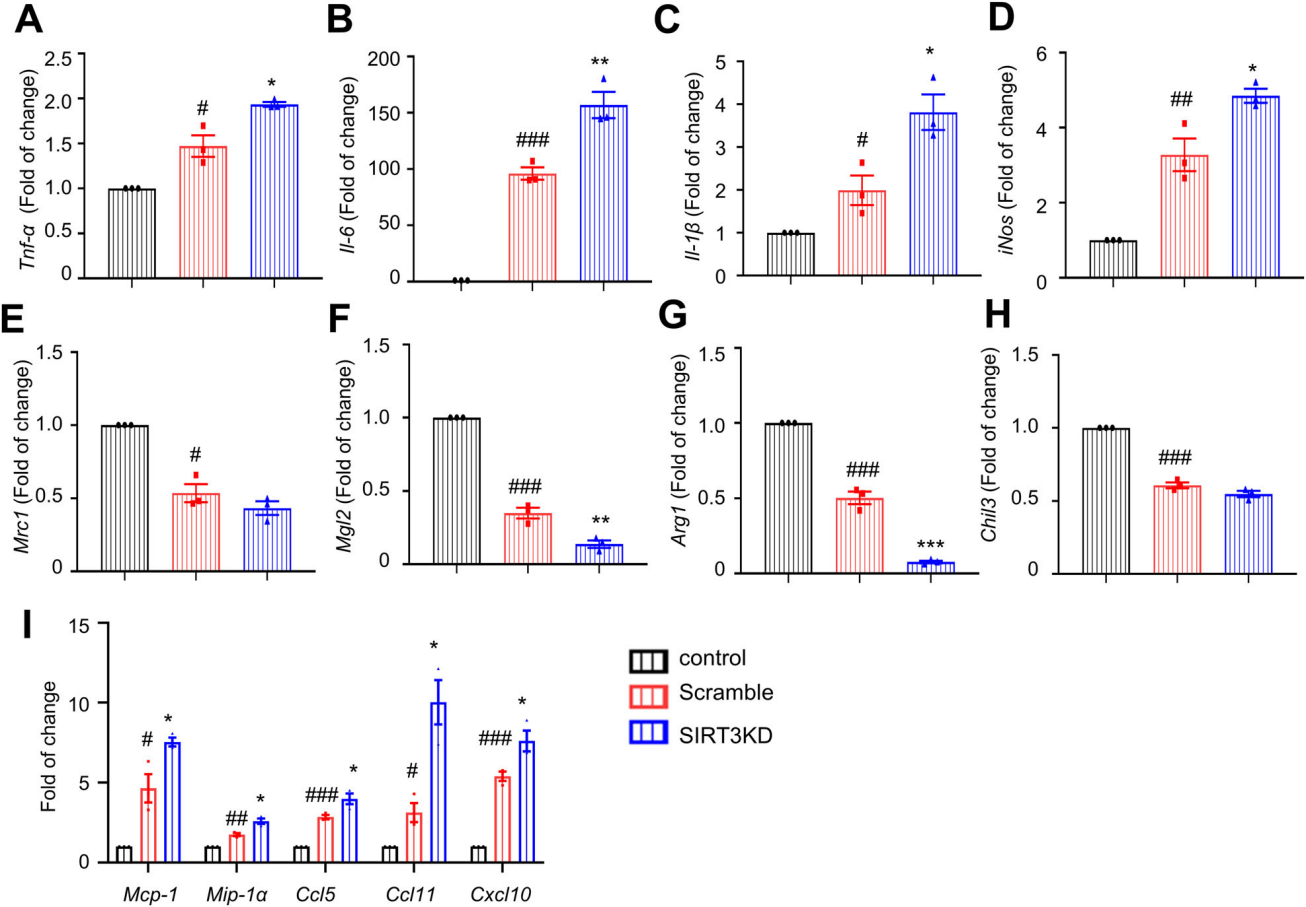
SIRT3 silencing aggravates adipocytes CM induced inflammatory responses in macrophages. The mRNA expression of *Tnf-α* (**A**) *Il-6* (**B**) *Il-1β* (**C**) and *iNos* (**D**) in BMDMs. The mRNA expression of *Mrc1* (**E**) *Mgl2* (**F**) *Arg1* (**G**) and *Chil3* (**H**) in BMDMs. **I** The mRNA expression of *Mcp-1*, *Mip-1α*, *Ccl5*, *Ccl11* and *Cxcl10* in BMDMs. *n* = 3 independent experiments. Data are expressed as means ± SEM. ^#^*P* < 0.05, ^##^*p* < 0.01, ^###^*p* < 0.001, Scramble vs. control; ^*^P < 0.05, **P < 0.01, ***P < 0.001, Scramble vs. SIRT3KD.

It has been reported that adipocytes secrete a series of molecules to regulate inflammatory responses in macrophages [44]. Therefore, we recruited an indirect co-culture system to investigate the effects of adipocyte-expressed SIRT3 in mediating the crosstalk between macrophages and adipocytes. We generated SIRT3 overexpressed 3T3-L1 adipocytes (SIRT3OE), with about three-fold higher SIRT3 protein expression (Fig. S2A). CM were harvested from both vector and SIRT3OE adipocytes. When incubated in CM from the vector adipocytes, the mRNA expression of pro-inflammatory macrophage markers, including *Tnf-α*, *Il-6*, *Il-1β* and inducible nitric oxide synthase (*iNos*), were significantly higher (Fig. 3A‒D), and the mRNA expression of anti-inflammatory macrophage markers, including mannose receptor C-type 1 (*Mrc1*), macrophage Gal/GalNAc lectine 2 (*Mgl2*), arginase 1 (*Arg1*) and chitinase-like 3 (*Chil3*), were greatly lower (Fig. 3E‒H) in BMDMs, as compared to those from DMEM-incubated cells. While, incubation in CM from SIRT3OE adipocytes totally reversed the increases of pro-inflammatory macrophage markers and decreases of anti-inflammatory macrophage markers (Fig. 3A‒H). Furthermore, the flow cytometry results showed that less F4/80/CD11c double-positive and slightly more F4/80/CD206 double-positive BMDMs were detected after incubation in CM from the SIRT3OE adipocytes, from compared with that of the incubation in CM from the vector adipocytes (Fig. 3I). Additionally, the adipocyte CM-induced upregulation of *Mcp-1*, *Mip-1α*, *Ccl5*, *Ccl11*, *Cxcl10* and *Cxcl11* mRNA expression in BMDMs was reversed by the CM from the SIRT3OE cells (Fig. 3J), indicating that SIRT3 overexpression in adipocytes attenuated macrophage chemotaxis. Next, we recruited transwell system to confirm the effect of adipocyte-expressed SIRT3 in macrophage migration. When co-cultured with SIRT3OE adipocytes, the number of migrated BMDMs was markedly decreased, compared with that of the vector cells (Fig. 3K). To further confirm the above results, siRNA-mediated *SIRT3* knockdown (SIRT3KD) 3T3-L1 cells was generated (Fig. S2B). CM were harvested from scramble and SIRT3KD adipocytes, respectively. When incubated in CM from SIRT3KD adipocytes, the mRNA expression of pro-inflammatory macrophage markers, including *Tnf-α*, *Il-6*, *Il-1β* and *iNos*, were significantly higher (Fig. 4A‒D), and the mRNA expression of anti-inflammatory macrophage markers, including *Mrc1*, *Mgl2*, *Arg1* and *Chil3*, were greatly lower (Fig. 4E‒H) in BMDMs, as compared to those from the scramble adipocytes. Additionally, the mRNA expression of *Mcp-1*, *Mip-1α*, *Ccl5*, *Ccl11* and *Cxcl10* (Fig. 4I) in BMDMs was increased when treated with CM from SIRT3KD cells. Taken together, these results suggested that adipocyte-expressed SIRT3 attenuates the chemotaxis and pro-inflammatory polarization of macrophages.

### SIRT3 overexpression alters metabolic profiles of 3T3-L1 adipocytes

**Fig. 5:**
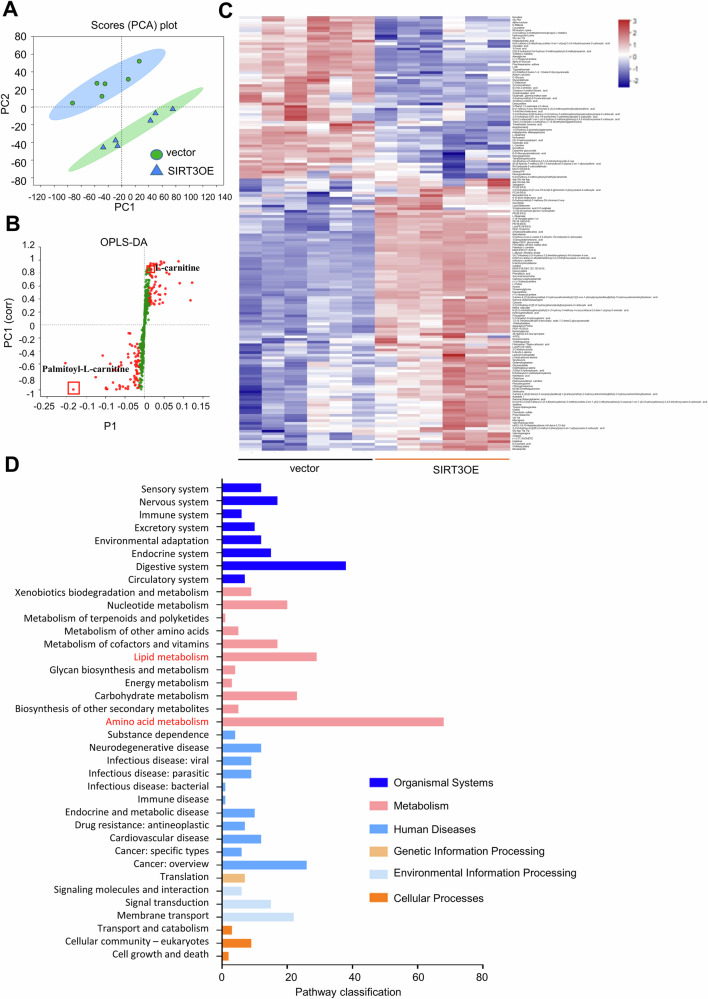
Metabolomic profiles of CM from vector and SIRT3OE adipocytes. **A** PCA analysis of vector and SIRT3OE cells. Each dot represents the technological replicate analysis of samples. **B** S-plot generated from OPLS-DA. Dot represents metabolites, which are p [1] < -0.05 or > 0.05 and p (corr) [1] < -0.5 or > 0.5 and marked in red dot. (**C**) Heat maps of significant differentially expressed metabolites. (**D**) Pathway classifications were selected to plot. *n* = 6 replicates.

Cellular metabolites are considered as important regulators for macrophage chemotaxis and polarization [45]. To decipher the metabolites mediating the crosstalk between adipocytes and macrophages, metabolomics analysis of the CM from the vector and SIRT3OE adipocytes was performed. Herein, six biological replicates of each group yielded 12 data points. To reflect the difference of metabolites in CM from the vector and SIRT3OE adipocytes, the recognition of the sample patterns was determined by orthogonal partial least-squares discriminant analysis (OPLS-DA), followed by ranking the altered metabolites in loading. The vector and SIRT3OE groups formed two separate clusters in this model, demonstrating the separation of two groups (Fig. 5A).

The differential altered metabolites (*P* < 0.05 in *t*-test, VIP > 1 in OPLS-DA and fold change > 2) were deciphered to investigate the influence of SIRT3 on the metabolic pathways. The abundance of 166 metabolites in the SIRT3OE adipocytes was identified to be different when compared with the vector cells (Fig. 5B). These metabolites were identified and annotated in Table S3, and further displayed in the clustering heatmap (Fig. 5C). The biological categories of the identified metabolites were retrieved by Kyoto Encyclopedia of Genes and Genomes (KEGG). The top two abundant changed metabolic pathways were amino acid metabolism and lipid metabolism (Fig. 5D).

**Fig. 6:**
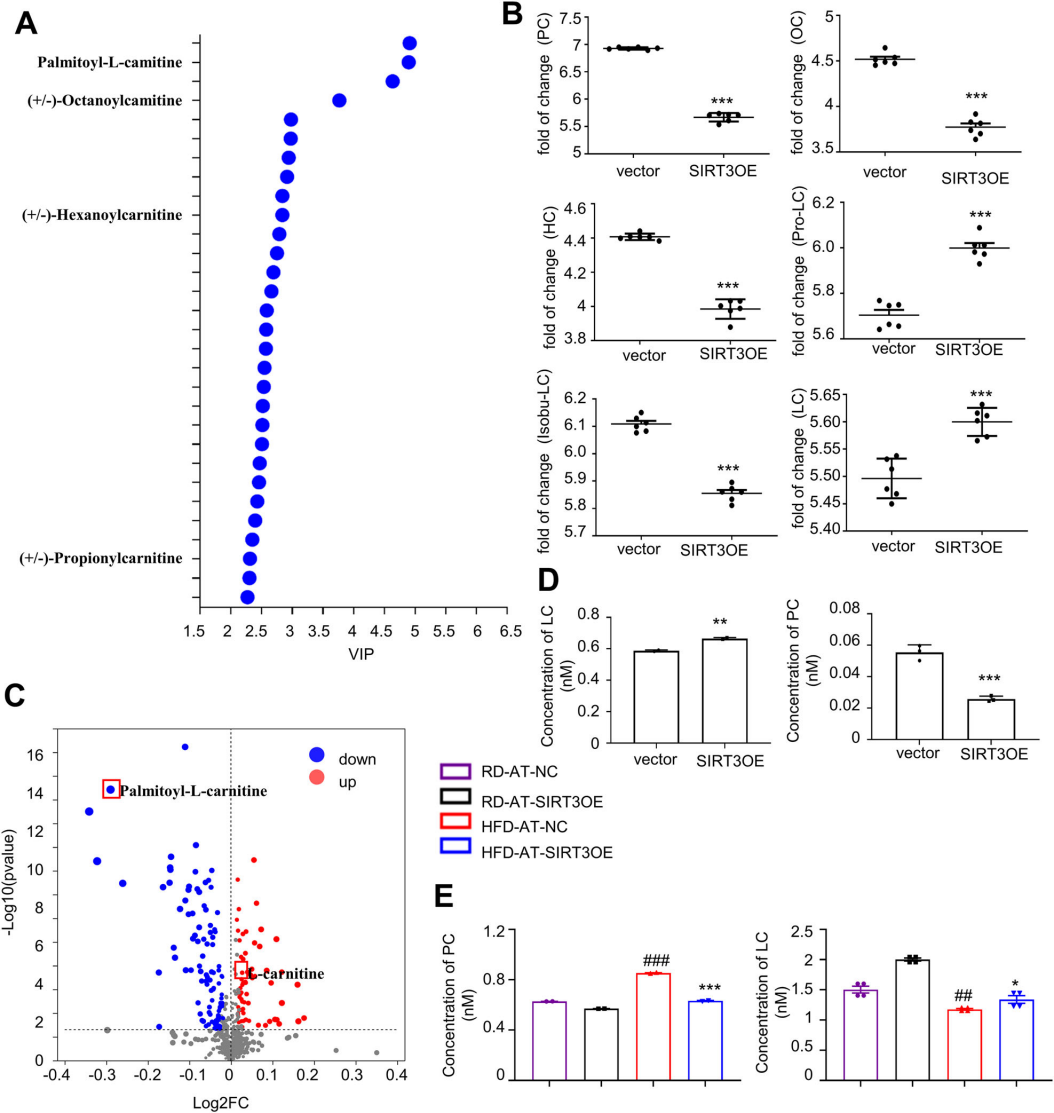
SIRT3 increases LC content and decreases acylcarnitines contents in CM of adipocytes. **A** VIP of significantly altered metabolites. **B** Scatter diagram of PC, OC, HC, Pro-LC, Isobu-LC and LC, each dot shows a technical replicate. **C** Volcano plot of significantly altered metabolites between the vector and SIRT3OE cells. *n* = 6 replicates. **D** Contents of PC and LC in CM of vector and SIRT3OE adipocytes. *n* = 6 replicates. ^*^P < 0.05, **P < 0.01, ***P < 0.001, vector vs. SIRT3OE. **E** Contents of PC and LC in serum of mice. *n* = 4 mice per group. Data are expressed as means ± SEM. ^##^*P* < 0.01, ^###^*P* < 0.001, HFD-AT-NC vs. RD-AT-NC; *P < 0.05, **P < 0.01, ***P < 0.001, HFD-AT-NC vs. HFD^-^AT-SIRT3OE.

As shown in Fig. 6A, the substances with the top thirty VIP values include palmitoyl-L-carnitine (PC), octanoyl-L-carnitine (OC), hexanoyl-L-carnitine (HC) and propionylcarnitine (Pro-LC). Acylcarnitines are formed from the conjugation of FAs with LC [46]. The levels of LC and Pro-LC were increased, while the levels of other acylcarnitines, including PC, OC, HC and isobutyryl carnitine (Isobu-LC), were decreased in CM from the SIRT3OE adipocytes, compared with those in CM from the vector adipocytes (Fig. 6B). Among the differential metabolites, 104 metabolites were decreased, and 62 metabolites were increased in the SIRT3OE group, as displayed in the volcano plot (Fig. 6C). PC showed the greatest correlations and covariance in the predictive component between the vector and the SIRT3OE cells (Fig. 6A, C). Furthermore, UPLC-MS/MS analysis indicated that LC was accumulated while PC was depleted in SIRT3OE adipocytes compared to the vector cells (Fig. 6D). Additionally, the serum level of LC was higher, and the serum level of PC was lower in AT-SIRT3OE mice, compared with those of AT-NC mice (Fig. 6E). Taken together, SIRT3 overexpression greatly alters the metabolic profiles in adipocytes, accompanied with accumulation of LC.

### Exogenous LC suppresses pro-inflammatory polarization of BMDMs

**Fig. 7:**
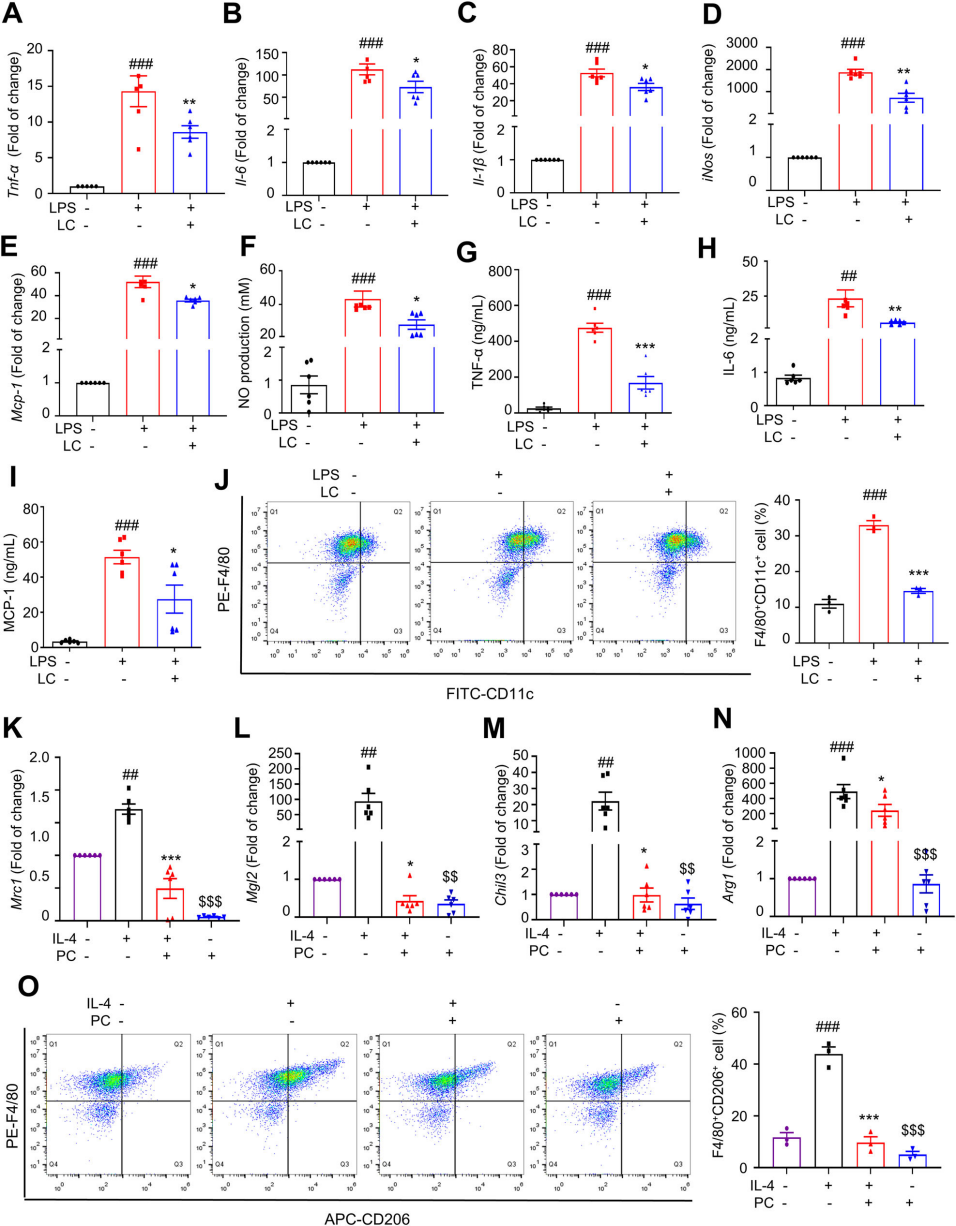
Exogenous LC suppresses pro-inflammatory polarization of macrophages, while exogenous PC suppresses anti-inflammatory polarization of macrophages. DMDMs were cultured in the presence or absence of LC for 12 h. The mRNA expression of *Tnf-α* (**A**) *Il-6* (**B**) *Il-1β* (**C**), *iNos* (**D**) and *Mcp-1* (**E**) in BMDMs. The secretion of NO (**F**) TNF-α (**G**) IL-6 (**H**) and MCP-1 (**I**) in the culture supernatant from BMDMs. **J** The F4/80/CD11c double positive macrophages were analyzed by flow cytometry. ^##^*P* < 0.01, ^###^*P* < 0.001, LPS vs. control; ^*^P < 0.05, ^**^P < 0.01, ^***^P < 0.001, LPS vs^.^ LPS + LC. DMDMs were cultured in the presence or absence of PC 12 h. The mRNA expression of *Mrc1* (**K**) *Mgl2* (**L**) *Ym1* (**M**) and *Arg1* (**N**) in BMDM. *n* = 6 independent experiments. **O** The F4/80/CD206 double positive macrophages were analyzed by flow cytometry. *n* = 3 independent experiments. ^##^*P* < 0.01, ^###^*P* < 0.001, IL-4 vs. control; *P < 0.05, ***P < 0.001, IL-4 vs. IL-4 + PC. ^$$^*P* < 0.01, ^$$$^*P* < 0.001, PC vs. control. Data are expressed as means ± SEM.

LC was demonstrated with anti-inflammatory property [17, 47]. LC showed no cytotoxicity on BMDMs up to 100 μM (Fig. S3A). LPS plus IFN-γ treatment remarkably increased the mRNA levels of *Tnf-α*, *Il-6*, *Il-1β*, *iNos* and *Mcp-1* in BMDMs, whereas 100 µM LC reversed the increased levels of the cytokines (Fig. 7A‒E). The anti-inflammatory effect of LC was corroborated by observing a consistent reduced releases of the pro-inflammatory mediators, including NO, TNF-α, IL-6 and MCP-1 (Fig. 7F‒I). Remarkably, LPS plus IFN-γ treatment induced more F4/80/CD11c double-positive cells, while LC treatment decreased the percentage of F4/80/CD11c double-positive cells (Fig. 7J). In summary, these results demonstrated that LC suppresses pro-inflammatory polarization in BMDMs.

### Exogenous PC suppresses anti-inflammatory polarization in BMDMs

LCACs show the potential to activate pro-inflammatory responses in macrophages [33, 48]. PC showed no cytotoxicity on BMDMs up to 100 μM (Fig. S3B). IL-4 treatment remarkably upregulated the mRNA levels of anti-inflammatory macrophage markers in BMDMs, including *Mrc1*, *Mgl2*, *Chil3* and *Arg1*, whereas 50 µM PC reversed the increases of these markers (Fig. 7K‒N). Remarkably, IL-4 treatment showed more F4/80/CD206 double-positive cells, while PC reversed the change (Fig. 7O). Taken together, PC suppresses polarization of BMDMs towards an alternative anti-inflammatory activation state.

### Exogenous PC and LC manipulate FAO in BMDMs

**Fig. 8:**
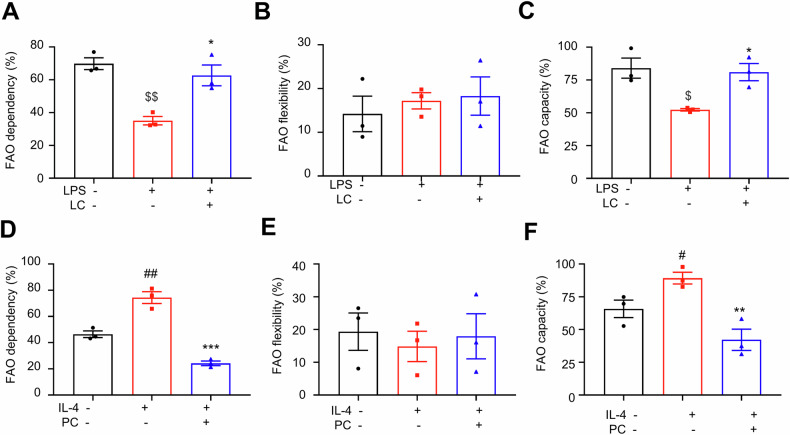
Exogenous PC and LC regulate FAO in BMDMs. DMDMs were cultured in the presence or absence of LC for 12 h. FAO dependency (**A**), flexibility (**B**) and capacity (**C**) were analyzed by Seahorse mitochondrial fuel flex test. ^$^*P* < 0.05, LPS vs. control. ^$$^*P* < 0.01, LPS vs. control. ^*^P < 0.05, LPS vs. LPS + LC. DMDMs were cultured in the presence or absence of PC 12 h. FAO dependency (**D**), flexibility (**E**) and capacity (**F**) were analyzed by Seahorse mitochondrial fuel flex test. *n* = 3 replicates. Data are expressed as means ± SEM. ^##^*P* < 0.01, IL-4 vs. control, ^**^P < 0.01, IL-4 vs. IL-4 + PC, ^***^P < 0.001, IL-4 vs. IL-4 + PC.

Pro-inflammatory and anti-inflammatory macrophages exhibit different metabolic characteristics [8]. LC participates in the shift of long-chain FAs from the outside to inside of mitochondrial membrane and functions as a cofactor for β-oxidation of FAs [49]. To assess the potential roles of LC and PC in manipulating FAO in BMDMs, we determined the dependency, capacity, and flexibility of BMDMs for long-chain FAs by measuring the OCR in the presence or absence of inhibitors of three fuel pathways. LPS plus IFN-γ treated BMDMs exhibited lower dependency (Fig. 8A), no change of flexibility (Fig. 8B) and slightly lower capacity (Fig. 8C) on FAO, indicated M1 BMDMs did not rely on FAO to maintain baseline respiration. Surprisingly, LC obviously enhanced the FAO dependency (Fig. 8A). In addition, IL-4-treated BMDMs showed increased FAO dependency and capacity, but not FAO flexibility, compared with the control cells (Fig. 8D‒F), indicating that IL-4 positively promoted FAO in BMDMs. Interestingly, PC treatment showed a trend towards reduced FAO dependency and capacity (Fig. 8D, F), which suggested that PC blocked FAO in BMDMs. In summary, LC and PC manipulate the dependency and capacity of FAO in BMDMs, which may contribute to macrophage polarization.

### SIRT3 decreases the accumulation of PC in adipocytes through deacetylating CPT2

**Fig. 9:**
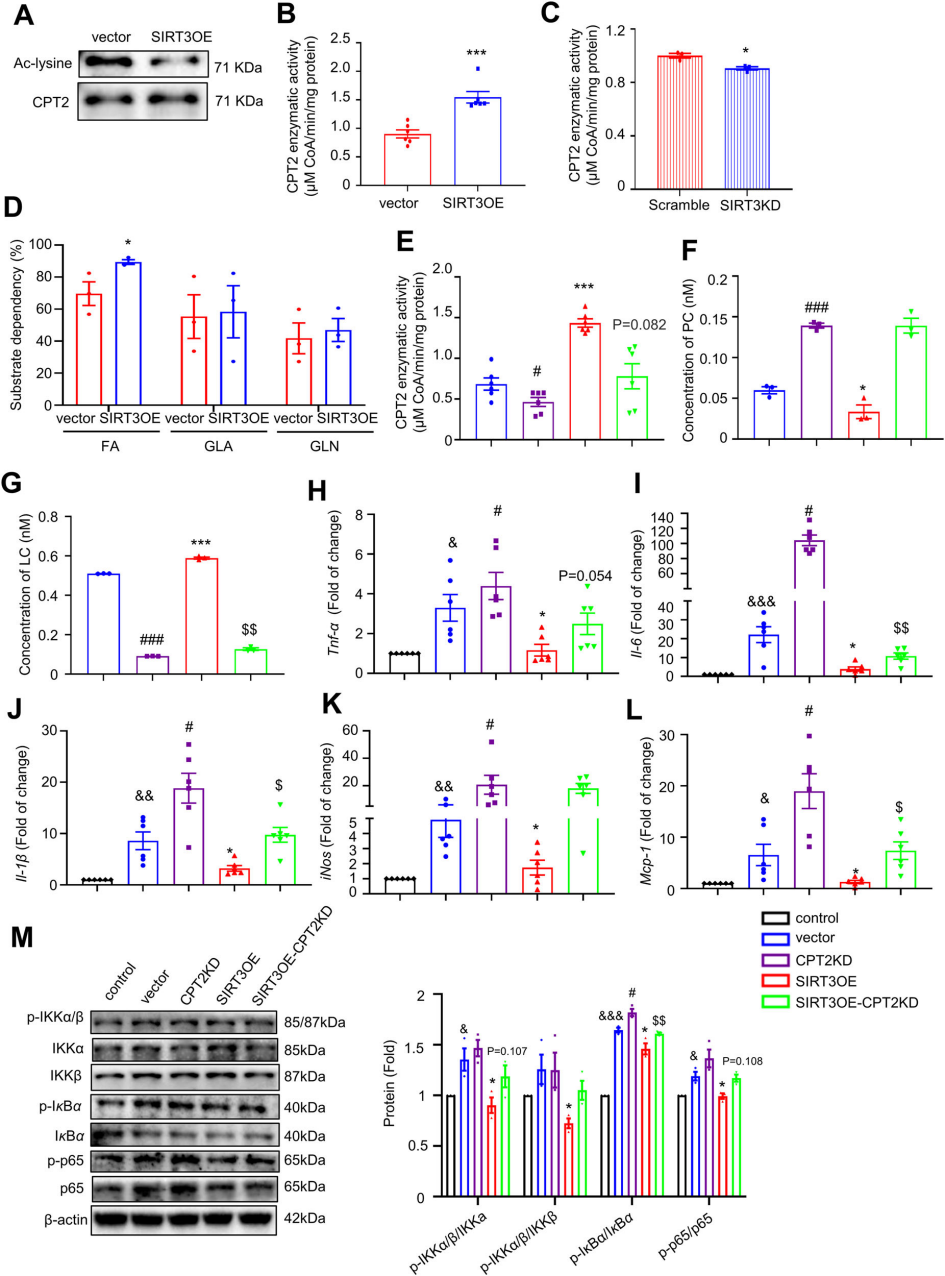
SIRT3 decreases the accumulation of PC through deacetylating CPT2. **A** The acetylated CPT2 levels in vector and SIRT3OE adipocytes. *n* = 3 independent experiments. **B** The CPT2 activity in vector and SIRT3OE adipocytes. *n* = 6 replicates. **C** The CPT2 activity in Scramble and SIRT3KD adipocytes. *P < 0.05, Scramble vs. SIRT3KD. *n* = 3 replicates. **D** The substrate dependency in vector and SIRT3OE adipocytes was determined by Seahorse XF mitochondrial fuel flex test. *n* = 3 replicates. **E** The CPT2 activity was evaluated. *n* = 6 replicates. The contents of PC (**F**) and LC (**G**) in CM of adipocytes. *n* = 3 replicates. The mRNA expression of *Tnf-α* (**H**), *Il-6* (**I**), *Il-1β* (**J**), *iNos* (**K**) and *Mcp-1* (**L**) in BMDMs treated with CM of adipocytes. *n* = 6 replicates. **M** The protein expression of p-IKKα/β, IKKα, IKKβ, p-IκBα, IκBα, p-p65 and p65. *β*-actin was used as a loading control. *n* = 3 replicates. Data are expressed as means ± SEM. *P < 0.05, **P < 0.01, ***P < 0.001, vector vs. SIRT3OE; ^#^*P* < 0.05, ^##^*P* < 0.01, ^###^*P* < 0.001, vector vs. CPT2KD; ^&^*P* < 0.05, ^& &^*P* < 0.01, control vs vector; ^$^*P* < 0.05, ^$$$^*P* < 0.001, CPT2KD vs. SIRT3OE-CPT2KD.

CPT2, located on the inner mitochondrial membrane, converts LCACs into LC and long-chain acyl-coenzyme As (acyl-CoAs) to facilitate FAO. SIRT3 directly deacetylated CPT2 to reduce the acetylated form of CPT2 (Fig. 9A) and SIRT3 overexpression enhanced its enzymatic activity (Fig. 9B) in adipocytes. Whereas, SIRT3 silencing suppressed CPT2 enzymatic activity in adipocytes (Fig. 9C). Next, the mitochondrial fuel dependency tests were performed in the vector and the SIRT3OE adipocytes, achieved by co-treatment of inhibitors of mitochondrial pyruvate carrier (glucose oxidation pathway), glutaminase (glutamine oxidation pathway) and CPT1 (FAO pathway) successively. As shown in Fig. 9D, the dependency of long-chain FAs oxidation was obviously higher in SIRT3OE adipocytes than that in the vector cells, but not pyruvate or glutamine, suggesting that SIRT3OE cells can switch their metabolic phenotype to be more reliant on FAO. Altogether, these results indicated that SIRT3 mainly enhances FAO in adipocytes.

Recent studies underscored that deficiency of CPT2 causes the increase of LCACs, especially C16 and C18 acylcarnitine [30]. To further confirm the above results, CPT2 was silenced in both vector and SIRT3OE adipocytes (Fig. S4). The enzymatic activity of CPT2 was greatly decreased in both CPT2KD and SIRT3OE-CPT2KD adipocytes (Fig. 9E). Moreover, compared with the CM from the vector adipocytes, less PC and more LC were detected in CM from the SIRT3OE adipocytes, which were totally abolished in CM from the SIRT3OE-CPT2KD cells (Fig. 9F, G). In summary, SIRT3 enhances FAO by deacetylating and activating CPT2, which subsequently alters the relative content of LC and PC in adipocytes.

To further confirm the effect of CPT2 silencing on the crosstalk between adipocytes and macrophages, macrophage-adipocyte co-culture was performed. Similarly, CM from the SIRT3OE adipocytes obviously reduced the mRNA expression of pro-inflammatory macrophage markers (*Tnf-α*, *Il-6*, *Il-1β*, *iNos* and *Mcp-1*), when compared with CM from the vector adipocytes (Fig. 9H‒L). Surprisingly, compared with the CM from the vector adipocytes, CM from the CPT2KD adipocytes remarkably upregulated the mRNA expression of pro-inflammatory macrophage markers in BMDMs, whereas CM from the SIRT3OE-CPT2KD adipocytes partially reversed these changes (Fig. 9H‒L). The nuclear factor-κB (NF-κB) signaling pathway is a central regulator orchestrating inflammatory activation [50]. Thus, the NF-κB signaling pathway was investigated. As shown in Fig. 9M, incubation with CM from the vector adipocytes enhanced the phosphorylation of IKKα/β, IκBα, and p65, suggesting activation of NF-κB signaling pathway, which was partially reversed by CM from the SIRT3OE-CPT2KD adipocytes. Taken together, adipocyte-expressed SIRT3 orchestrates BMDM polarization through regulating CPT2 activity and suppressing NF-κB signaling pathway.

## Discussion

Mounting evidence implicates that obesity represents a state of long-term, low-grade inflammation, and ATMs are a significant contributor to systematic inflammation and dysfunctional insulin action in obese individuals [51]. The utilization of FAs, especially FAO, is vital to understand the contribution of lipid metabolism to inflammation, in contrast to lipotoxicity in the case of lipid overload and excessive accumulation [52]. SIRT3 has been implicated in various metabolic-related diseases, through directly deacetylating multiple enzymes in mitochondrial metabolism [53]. In addition, recent studies have indicated that SIRT3 deficiency exacerbated pro-inflammatory cytokine production [54, 55]. Herein, we uncovered that AT expressed SIRT3 reduces macrophage infiltration and prevents macrophage polarization towards pro-inflammatory state, thus attenuating systemic inflammation and improving insulin sensitivity in HFD feeding environments. Considering lower SIRT3 expression/activity and more pronounced inflammation in AT of obese patients, these findings have significant clinical implications. Enhancing SIRT3 level and/or mimicking its activity in AT could offer hope for more effective and targeted interventions to ameliorate systematic inflammation and combat obesity-related metabolic diseases. While direct SIRT3 activators are not yet clinically available, recent studies supported that SIRT3 activators reduced chronic inflammation and improved IR, such as berberine [36], honokiol [56] and guttiferone J [57]. Therefore, effective SIRT3 activators targeting AT could be promising drug candidates against obesity-associated metabolic disorders.

ATMs are strongly associated with AT metabolism and endocrine homeostasis [58]. A growing body of data evidence suggested that the arrangement of macrophages in a crown-like structure (CLS) around dying adipocyte exhibits features of necrosis in obese subjects [59]. The pro-inflammatory cytokines are excessively secreted from WAT in either genetic-based or diet-induced obese mice, which in turn interrupt insulin action in adipocytes, contributing to the onset and development of insulin resistance [60]. In HFD-fed mice, AT-SIRT3OE mitigated the secretion of inflammatory cytokines and chemokines from eWAT, thereby improving systemic inflammation and insulin sensitivity.

The polarization from anti-inflammatory type to pro-inflammatory type macrophages is thought to contribute to AT inflammation in obesity [61]. Interestingly, pro-inflammatory macrophages rely more on aerobic glycolysis, while anti-inflammatory macrophages are known to be more dependent on OXPHOS [9]. SIRT3 is indispensable for maintaining mitochondrial protein acylation homeostasis, and thereby cell metabolism, through deacetylating key enzymes in various metabolic pathways [28, 53]. SIRT3 manipulates mitochondrial FAO by regulating the activity of LCAD in hepatic mitochondria [25, 29], and CPT2 in hepatic cell [28] and platelet [30]. In the current study, SIRT3 attenuates adipocytes CM induced inflammatory responses in BMDMs. A pertinent question arising from our findings is how SIRT3 facilitates macrophage phenotype transition through crosstalk with adipocytes.

Acylcarnitines are esters produced by the conjugation of FAs with LC, playing important roles in many cellular energy metabolism [48]. Mitochondrial long-chain FA β-oxidation requires the involvement of several enzymes, including carnitine acyltransferases, to enable the translocation of acyl-CoAs from the cytoplasm into the mitochondrial matrix [62]. A human genetic disease characterized by CPT2 deficiency leads to elevated acylcarnitine levels in serum caused by impaired FAO. Acylcarnitines accumulation was considered a character of CPT2 dysfunction [63]. Plasma LCAC species were also increased in both obesity and type 2 diabetes subjects compared to lean ones [32, 64]. Additionally, the plasma and liver LCACs were accumulated in SIRT3 knockout mice [29]. Consistently, we found that LC was accumulated and PC was depleted in the CM from SIRT3OE adipocytes. LC accumulation indicated the possibility of enhanced CPT2 activity, since CPT2 converts acylcarnitine back into coenzyme A ester and LC. As expected, SIRT3 overexpression results in less acetylated CPT2, thereby activating CPT2 and altering the metabolism of LC and PC. The positive effect of SIRT3 was not completely eliminated by CPT2 silencing, which suggested other targets might also mediate the benefit role of SIRT3.

Until now, research on immune cells primarily focused on FAO, rather than the potential role of FA metabolites in immune cell functioning, such as acylcarnitines. Besides adipokines and cytokines, adipocyte-derived molecules, such as lactate [65] and glutamine [66], function as signaling mediators to facilitate the interaction between adipocytes and ATMs [67]. It is intriguing to explore the role of acylcarnitines in manipulating the immune cell responses. Previous studies demonstrated that acylcarnitines activate pro-inflammatory signaling pathways, resulting in undesirable consequences of dysregulated FAO in mitochondria [33, 46, 68]. Additionally, LC might act as a protective molecule in the tissue destruction in inflammation [69]. Previous studies found that mitochondrial respiration and FAO are related with IL-4-induced macrophage polarization [18]. Our studies found that PC altered the metabolic phenotypes of IL-4-induced BMDMs, less dependency on FAO, which disclosed an essential role of adipocyte-derived LC and PC in manipulating the AT inflammatory microenvironment.

In summary, our study uncovered a novel role of adipocyte-expressed SIRT3 in mitigating infiltration and pro-inflammatory polarization of macrophages, thereby attenuating AT inflammation and protecting against obesity-related insulin resistance. Through deacetylating CPT2, SIRT3 alters cellular metabolic phenotype to be more reliant on FAO, thereby contributing to the accumulation of LC and reduction of PC. Through paracrine mode, PC and LC alter macrophage polarization by regulating mitochondrial OXPHOS levels of FAs. These findings further highlight that the crosstalk between adipocytes and macrophages plays a central role in maintaining AT homeostasis. Therapeutic interventions that either promote SIRT3 activity or enhance LC secretion in adipocytes could be a promising strategy to treat obesity-associated insulin resistance and metabolic disorders.

## Supplementary information

Supplemental material

Supplemental Table S3

original Western blots for reviewers

## Data Availability

All datasets are available from the corresponding author on reasonable request.
