# Supplementary material for: Adipocyte-expressed SIRT3 manipulates carnitine pool to orchestrate metabolic reprogramming and polarization of macrophages
Source: Cell Death Dis. 2025 May 15;16(1):381. doi: 10.1038/s41419-025-07699-6 (PMC12078679; doi:10.1038/s41419-025-07699-6)
Supplement: Supplementary file 1 — Supplemental material [file 41419_2025_7699_MOESM1_ESM.pdf]

Adipocyte-expressed SIRT3 manipulates carnitine pool to orchestrate metabolic reprogramming and polarization of macrophages

Running title: Adipocyte SIRT3 mitigates adipose tissue inflammation

Jiali Chen[1,2†], Fei Zhou[1†], Lei Zhang[1†], Ruohan Lou[1], Cangman zhang[1], jianbo Wan[1], Xiaojun Ma[3*]and Ligen Lin[1,4]*

[1] State Key Laboratory of Quality Research in Chinese Medicine, Institute of Chinese Medical Sciences, University of Macau, Macao.

[2] Guangdong Provincial Key Laboratory of Medical Immunology and Molecular Diagnostics, Guangdong Medical University, Dongguan 523808, China.

[3] The department of Endocrinology and Metabolism, The First Affiliated Hospital of Zhengzhou University, Zhengzhou 450001, China.

[4] Department of Pharmaceutical Sciences and Technology, Faculty of Health Sciences, University of Macau, Macao.

[†] Jiali Chen, Fei Zhou and Lei Zhang contributed equally to this work.

***Correspondence**

Xiaojun Ma. Address: No.1 Jianshe East Road, Erqi District, Zhengzhou City, Henan, China. email: fccmaxj1@zzu.edu.cn.

Ligen Lin. Address: 6044, N22, University of Macau, Avenida da Universidade, Taipa, Macao. email: ligenl@um.edu.mo.

Supplementary Methods

HOMA-IR

Serum insulin was measured using the insulin assay kit according to the manufacturer's instructions. The HOMA-IR index was calculated as following: fasting serum glucose × fasting serum insulin/14.1, to assess insulin resistance [1].

GTTs and ITTs

GTTs and ITTs were performed as described previously [2]. GTTs were carried out after fed with RD or HFD for 11 weeks. After 16 h fasting, the basal blood glucose level was measured using the OneTouch Ultra blood glucose meter and LifeScan test strips. Subsequently, the mice were intraperitoneally injected with a glucose (Sigma-Aldrich) solution (1.5 g/kg body weight). The tail blood glucose was measured at 15, 30, 60, 90, and 120 min after injections. ITTs were performed after fed with RD or HFD for 12 weeks. After 6 h fasting, the basal blood glucose level was measured. Subsequently, the mice were administered by an intraperitoneal injection of human insulin (Eli Lilly, Indianapolis, IN, USA) at a dose of 1.0 U/kg body weight. Then, the tail blood glucose was measured at 15, 30, 60, 90, and 120 min after injections.

Determination of cytokines

The cytokines in cell media, mice sera and tissue lysates were determined using commercial ELISA kits (Neobioscience Technology Co., Ltd., Shenzhen, China), following the manufacturer's instruction. Nitric oxide (NO) production in cell medium was determined using Griess reagent (Sigma-Aldrich).

Histological analysis

The eWAT was fixed in 4% buffered formalin, and then embedded in paraffin. H&E staining, Manson's trichrome staining, and Sirius Red staining were performed according to standard experimental procedures.

Immunohistochemistry

Immunohistochemical analysis of eWAT sections was performed as described previously [3]. Paraffin-embedded sections of eWAT were incubated with anti-F4/80, anti-CD11c and anti-CD206 antibodies at 4 °C overnight. The slides were incubated with the corresponding secondary antibodies for 2 h at room temperature. Histochemical reactions were performed using DAb as a substrate. Nuclei were counterstained with haematoxylin.

RNA interference and transient infection

The siRNA targeting SIRT3 (A: 5'-GGUGGGAGAAGGCCCAUAUTT-3; B: 5'-CAAGGUUCCUACUCCAUAUTT-3; C: 5'-GACCUUUGUAACAGCUACATT-3; D: 5'-GUCUGAAGCAGUACAGAAATT-3) were purchased from GenePharma (Shanghai, China). Scrambled non-targeting siRNA was used as a negative control. 3T3-L1 cells (1.5×10^5) were seeded at 6-well plates and differentiated into mature adipocytes. Mature 3T3-L1 cells were transfected with 50 nM siRNA using Lipofectamine 3 000 and OPTI-MEMI-reduced serum medium (Invitrogen) for 24 h. Cells were switched to fresh medium and used for further experiments.

Cell viability

Cell viability was determined by 3-(4,5-dimethyl-2-thiazolyl)-2,5-diphenyltetrazolium

bromide (MTT, Sigma-Aldrich) assay as described previously [4]. The BMDMs and 3T3-L1 preadipocytes were seeded in 96-well plates at a density of 1×10^4 cells per well. Cells were treated with different concentrations of LC or PC for 24 h. Then cells were incubated with DMEM containing MTT (1 mg/mL) for 4 h, followed by dissolving the formazan crystals with 100 μL DMSO. The absorbance at 570 nm was measured by a SpectraMax M5 microplate reader (Molecular Devices, CA, USA). The calculation equation for relative cell viability was as following: cell viability (%) = (As – A0)/(Ac – A0) × 100%, where As, A0 and Ac were the absorptions of test sample, blank control and negative control (DMSO).

Western blotting analysis

Western blotting was performed as described previously [5]. 3T3-L1 adipocytes, BMDMs and eWAT were lysed with RIPA lysis buffer (Beyotime Biotechnology, Shanghai, China). Protein concentration was determined using a BCA Protein Assay Kit (Thermo-Fisher). An equal number of proteins (20–30 μg) were separated by SDS-PAGE, transferred to PVDF membranes, blocked with 5% nonfat milk in TBST buffer (100 mM NaCl, 10 mM Tris-HCl, pH 7.5 and 0.1% Tween-20) for 2 h at room temperature, and incubated with specific primary antibodies (Table S1) overnight at 4 ºC. After washing with TBST thrice, a corresponding horseradish peroxidase conjugated secondary antibody was added and incubated for 2 h at room temperature. Signals were detected using a SuperSignal West Femto Maximum Sensitivity Substrate kit (Thermo-Fisher) and visualized using the ChemiDoc MP Imaging System (Bio-Rad, Hercules, CA, USA).

Real-time RT-PCR

Isolation of total RNA from cells and eWAT was performed using the TRIzol Reagent (Thermo-Fisher), following the manufacturer's instruction. 1 μg RNA was reversely transcribed into complementary cDNA using the SuperScript III First Strand Synthesis System (Thermo-Fisher). The qPCR experiments were conducted on a Step-One plus real-time PCR System using SYBR green PCR Master Mix (Thermo-Fisher) with gene specific primers (Table S2). The relative level of mRNAs was normalized to β-actin and calculated using the $2^{-\Delta\Delta Ct}$ method.

Co-immunoprecipitation

The indicated antibody was mixed with 20 μL protein A/G-Sepharose beads (Santa Cruz Biotechnology, CA, USA) and incubated at 4 ºC for 4 h. Cell lysates were added and incubated overnight at 4 ºC. After washed twice with RIPA, lysis buffer and sample preparation buffer were added. Samples were boiled for 5 min and then analyzed by Western blotting.

Metabolites extraction

The fully differentiated 3T3-L1 cells were incubated in DMEM supplied with 0.2% endotoxin and FA-free BSA for 48 h. Subsequently, the adipocytes culture medium was collected and centrifuged at 1,000 rpm for 10 min. The supernatant was harvested after centrifugation at 12,000 rpm at 4 °C for 10 min. 100 μL supernatant was mixed with 400 μL methanol:acetonitrile mix (1:1, v/v). The mixture was sonicated at 40 kHz at 5 °C for 30 min, placed at –20 °C for 30 min, and then centrifugated at 13,000 *g* at 4 °C for 15 min. The supernatant was carefully collected and evaporated to dryness under a gentle stream of nitrogen. The samples were dissolved in 100 μL acetonitrile:water (1:1, v/v). The solutions were spun for 15 min at 13,000 *g* at 4 °C and the cleared supernatants were used for UPLC-MS/MS analysis. Each sample contained six biological replicates.

UHPLC-MS/MS analysis

The instrument platform for UPLC-MS/MS analysis is UHPLC-Q Exactive system (Thermo Fisher Scientific). Chromatographic separation was performed using HSS T3 column (100 mm × 2.1 mm i.d., 1.8 μm). The mobile phases are consisted of 0.1% formic acid in water:acetonitrile (95:5, v/v) (solvent A) and 0.1% formic acid in acetonitrile:isopropanol:water (47.5:47.5:5, v/v) (solvent B). The gradient elution procedure was set as follows: from 0–0.1 min, 0–5% B; from 0.1–2 min, 5–25% B; from 2–9 min, 25–100% B; from 9–13 min, 100% B; from 13–13.1 min, 100–0% B; from 13.1–16 min, 0% B. The sample injection volume was 2 μL and the flow rate was set to 0.4 mL/min. The column temperature was maintained at 40 °C. During the period of analysis, all samples were stored at 4 °C. For mass spectrometer conditions, an electrospray ionization source (ESI) was used as the ion source, operating in either positive or negative ion mode. The optimal conditions were set as follows: heater temperature, 400 °C; capillary temperature, 320 °C; sheath gas flow rate, 40 arb; Aux gas flow rate, 10 arb; ion-spray voltage floating (ISVF), -2800 V in negative mode and 3500 V in positive mode, respectively; normalized collision energy, 20–40–60V rolling for MS/MS. Full MS resolution was 70000, and MS/MS resolution was 17500. Data acquisition was performed with the Data Dependent Acquisition (DDA) mode. The detection was carried out over a mass range of 70–1050 *m/z*.

Data preprocessing and annotation

The raw data of UPLC/MS/MS was preprocessed by Progenesis QI (Waters Corporation, Milford, USA) software, and a three-dimensional data matrix in CSV format was exported, including sample information, metabolite name and mass spectral response intensity. At the same time, the metabolites were searched and identified, and

the main database was the HMDB (http://www.hmdb.ca/), Metlin (https://metlin.scripps.edu/) and Majorbio Database.

The data after the database search was uploaded to the Majorbio cloud platform (https://cloud.majorbio.com) for data analysis.

Differential metabolites analysis

Perform variance analysis on the matrix file after data preprocessing. The R package ropls (Version 1.6.2) was used to perform principal component analysis (PCA) and orthogonal least partial squares discriminant analysis (OPLS-DA), and 7-cycle interactive validation was used to evaluate the stability of the model. In addition, student's t-test and fold difference analysis were performed. The selection of significantly different metabolites was determined based on the Variable importance in the projection (VIP) obtained by the OPLS-DA model and the p-value of student's t test, and the metabolites with VIP > 1, $p < 0.05$ were significantly different metabolites.

Differential metabolites among two groups were summarized, and mapped into their biochemical pathways through metabolic enrichment and pathway analysis based on database search (KEGG, http://www. genome.jp/kegg/). These metabolites can be classified according to the pathways they involved or the functions they performed.

Short name	tR (min)	Parent(m/z)	Daughters(m/z)	Collision energy(V)
LC	0.87	162.07	102.6	14
PC	9.37	260.15	84.81	22

References

1. Chen J, Feng Z, Zhou F, Lou R, Peng C, Ye Y*, et al.* 14-Deoxygarcinol improves insulin sensitivity in high-fat diet-induced obese mice via mitigating NF-kappaB/Sirtuin 2-NLRP3-mediated adipose tissue remodeling. Acta Pharmacol Sin. 2023;44:434-445.
2. Feng Z, Chen J, Chen C, Feng L, Wang R, Zhu J*, et al.* Bioactivity-based molecular networking-guided identification of guttiferone J from Garcinia cambogia as an anti-obesity candidate. British

journal of pharmacology. 2023;180:589-608.
3. Li D, Yang C, Zhu JZ, Lopez E, Zhang T, Tong Q, *et al.* Berberine remodels adipose tissue to attenuate metabolic disorders by activating sirtuin 3. Acta Pharmacol Sin. 2022;43:1285-1298.
4. Feng Z, Chen J, Feng L, Chen C, Ye Y, Lin L. Polyisoprenylated benzophenone derivatives from Garcinia cambogia and their anti-inflammatory activities. Food Funct. 2021;12:6432-6441.
5. Zhang T, Fang Z, Linghu KG, Liu J, Gan L, Lin L. Small molecule-driven SIRT3-autophagy-mediated NLRP3 inflammasome inhibition ameliorates inflammatory crosstalk between macrophages and adipocytes. Br J Pharmacol. 2020;177:4645-4665.

Table S1. Antibodies used for Western blotting.

Antibody	Source	Vendor	Catalog No.
anti-F4/80	Mouse	eBioscience	12-4801-82
anti-CD11c	Rabbit	ABclonal	A1508
anti-CD206	Mouse	BioLegend	141708
CPT2	Rabbit	proteintech	26555-1-AP
Acetylated-Lysine	Mouse	Cell Signaling Technology	# 9681
β-actin	Rabbit	cohesion bioscience	CPA9066
anti-SIRT3	Rabbit	Cell Signaling Technology	#5490
Anti-rabbit IgG		Cell Signaling Technology	#7074
Anti-Mouse IgG		Cell Signaling Technology	#7076
anti-phospho-NF-κB p65	Rabbit	Cell Signaling Technology	#3033
anti-NF-κB p65	Rabbit	Cell Signaling Technology	#8242
anti-phospho-IKKα/β	Rabbit	Cell Signaling Technology	#2697
anti-IKKα	Mouse	Cell Signaling Technology	#11930
anti-IKKβ	Rabbit	Cell Signaling Technology	#8943
anti-phospho-IκBα	Rabbit	Cell Signaling Technology	#2859
anti-IκBα	Mouse	Cell Signaling Technology	#4814

Table S2. List of oligonucleotide primers used in qRT-PCR.

Gene	Forward	Reverse
β-actin	AGGCCCAGAGCAAGAGAGGTA	GGGGTGTTGAAGGTCTCAAACA
Chil3	AGAAGGGAGTTTCAAACCTGGT	GTCTTGCTCATGTGTGTAAGTGA
Arg1	CTCCAAGCCAAAGTCCTTAGAG	AGGAGCTGTCATTAGGGACATC
Mgl2	TTAGCCAATGTGCTTAGCTGG	GGCCTCCAATTCTTGAAACCT
Mrc1	CAGGTGTGGGCTCAGGTAGT	TGTGGTGAGCTGAAAGGTGA
Il-1β	TGTTCTTTGAAGTTGACGGACCC	TCATCTCGGAGCCTGTAGTGC
Il- 6	CCAGAGATACAAAGAAATGATGG	ACTCCAGAAGACCAGAGGAAAT
iNos	CCAAGCCCTCACCTACTTCC	CTCTGAGGGCTGACACAAGG
Mcp-1	CAACTCTCACTGAAGCCAGCTC	TAGCTCTCCAGCCTACTCATTGG
Tnf-α	GAGAAAGTCAACCTCCTCTCTG	GAAGACTCCTCCCAGGTATATG
Ccl5	TGCAGAGGACTCTGAGACAGC	GAGTGGTGTCCGAGCCATA
Ccl11	AGAGCTCCACAGCGCTTCT	GCAGGAAGTTGGGATGGA
Cxcl10	GCTGCCGTCATTTTCTGC	TCTCACTGGCCCGTCATC
Mip-1α	CTTCTCTGTACCATGACACTCTGC	ATTCAGTTCCAGGTCAGTGATGTAT
Cxcl11	GGCTTCCTTATGTTCAAACAGGG	GCCGTTACTCGGGTAAATTACA
Cxcl12	TGCATCAGTGACGGTAAACCA	TTCTTCAGCCGTGCAACAATC

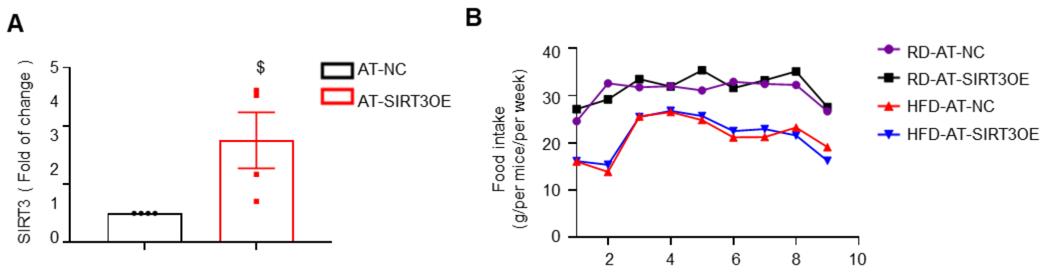

Fig. S1. (A) The mRNA expression of SIRT3 in eWAT from AT-NC and AT-SIRT3OE mice. (B) Food intake of mice with RD or HFD feeding. Data are expressed as means ± SEM. n=4–6. $^{*}p < 0.05$, AT-NC vs. AT-SIRT3OE.

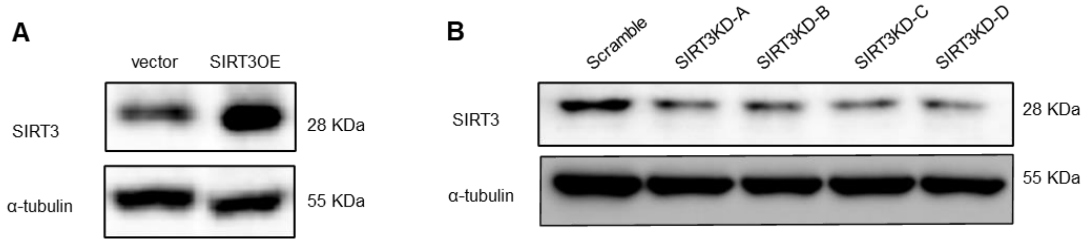

Fig. S2. (A) SIRT3 protein expression in the vector and SIRT3OE 3T3-L1 adipocytes. **(B)** SIRT3 protein expression in the scramble and SIRT3KD 3T3-L1 adipocytes.

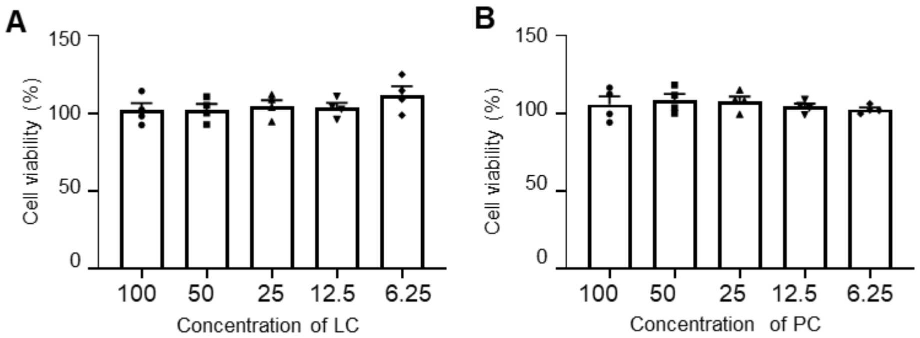

Fig. S3. Viability of BMDMs treated with different concentrations of LC or PC for 24 h, assessing by MTT assay. Data are expressed as means ± SEM. n=4.

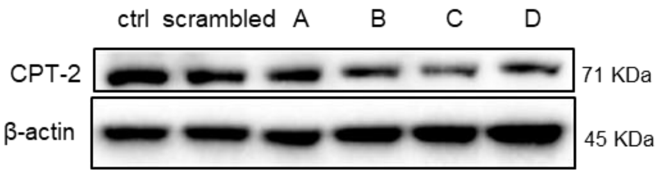

Fig. S4. The protein expression of CPT2 was detected in CPT2KD and scrambled 3T3-L1 cells.
