# Supplementary material for: Adipocyte-expressed SIRT3 manipulates carnitine pool to orchestrate metabolic reprogramming and polarization of macrophages
Source: Cell Death Dis. 2025 May 15;16(1):381. doi: 10.1038/s41419-025-07699-6 (PMC12078679; doi:10.1038/s41419-025-07699-6)
Supplement: Supplementary file 2 — Supplemental Table S3 [file 41419_2025_7699_MOESM2_ESM.pdf]

Metabolite	VIP pred OPLS-DA	VIP PLS-DA	FC(treated/control)	P value
PGF2alpha Alcohol methyl ether	2.9892	2.9301	0.9264	5.99E-14
Palmitoyl-L-carnitine	4.9077	4.8324	0.8181	3.78E-12
MG(0:0/20:1(11Z)/0:0)	4.9235	4.8371	0.7895	3.17E-11
Cytidine	2.6705	2.6183	0.9434	8.30E-10
5,6,7-trihydroxy-2-(4-hydroxy-3,5-dimethoxyphenyl)-4H-chromen-4-one	2.9257	2.7369	0.905	2.57E-09
L-NIL	2.1625	2.1462	1.0391	3.52E-09
MG(0:0/18:3(9Z,12Z,15Z)/0:0)	4.6459	4.5551	0.8	3.94E-09
6-[(2Z)-2-carboxy-2-ethylideneethoxy]-3,4,5-trihydroxyoxane-2-carboxylic acid	2.9851	2.8034	0.9036	7.30E-09
(+/-)-Hexanoylcarnitine	2.8487	2.7784	0.904	9.06E-09
Hypoxanthine	2.0097	1.9713	0.9686	9.67E-09
Alpha-CEHC glucuronide	2.2334	2.1161	0.9425	1.10E-08
Trigonellinamide	1.1687	1.1602	1.0114	2.37E-08
5-hydroxy-2-oxo-4-ureido-2,5-dihydro-1H-imidazole-5-carboxylate	1.7547	1.6576	0.9635	2.52E-08
Isobutyryl carnitine	2.1895	2.1439	0.9584	3.11E-08
3-Deoxyarabinohexonic acid	2.7997	2.6665	0.9028	3.20E-08
(+/-)-Octanoylcarnitine	3.7772	3.6771	0.8349	3.38E-08
PE(18:1(9Z)/0:0)	2.5232	2.3718	0.9326	4.62E-08
N-lactoyl-phenylalanine	2.9525	2.7814	0.893	4.91E-08
Beta-Alanine	1.6737	1.6022	0.9675	4.95E-08
L-Allysine Ethylene Acetal	2.2752	2.2266	0.9454	5.88E-08
Cinnamoylglycine	2.5858	2.5239	0.9316	6.38E-08
Deoxycytidine	2.4683	2.4147	0.9485	7.88E-08
Phenyllactic acid	2.546	2.3921	0.9268	1.79E-07
(S)-3-Hydroxyisobutyric acid	1.8689	1.7521	1.0436	2.34E-07
5,10-Pentadecadien-1-ol	1.8622	1.7673	0.9564	3.01E-07
Succinylmonocholine	2.8492	2.7897	0.9187	4.08E-07
Alpha-D-Glucose	1.2509	1.2065	1.0133	4.16E-07
Carboxycyclophosphamide	2.1889	2.1357	0.9591	4.40E-07
L-Proline	1.6942	1.651	0.9778	5.80E-07
L-Glutamate	2.2571	2.1954	0.938	6.31E-07
Inosine	2.3127	2.1568	0.9317	6.82E-07
L-Glutamine	1.0807	1.0523	1.01	1.17E-06
2-hydroxyhexadecanoic acid	1.8143	1.7253	0.9664	2.01E-06
Cytosine	2.3594	2.3186	0.9485	2.43E-06
(+/-)-Propionylcarnitine	2.3208	2.2982	1.0517	2.97E-06
2,3-Dimethyl-3-hydroxyglutaric acid	1.5931	1.5617	0.9732	3.03E-06
(E)-hex-2-enedioic acid	1.1493	1.1146	1.0119	3.36E-06
Norfuraneol	1.496	1.4021	1.0255	3.68E-06
Asparaginyl-Proline	1.4865	1.4859	0.9743	3.78E-06
PE(P-16:0e/0:0)	1.8937	1.7677	0.9664	4.00E-06
3-hydroxy-3-methyl-Glutaric acid	1.4218	1.3789	1.0202	4.61E-06
Methyl salicylate	2.2804	2.2434	0.9417	5.32E-06

PE(P-16:0/0:0)	1.8626	1.8336	0.9702	6.39E-06
3,4,5-trihydroxy-6-{[2-(3-hydroxyphenyl)acetyl]oxy}oxane-2-carboxylic acid	2.4398	2.3979	0.9386	7.29E-06
Isomaltose	2.48	2.3064	1.0789	7.66E-06
Myristoylglycine	1.9027	1.7925	0.9557	7.69E-06
2-Furanmethanol	1.1956	1.1626	1.0145	8.63E-06
N-[2-(3,4-dimethoxyphenyl)ethyl]-3-(1-hydroxy-3-methoxy-4-oxocyclohexa-2,5-dien-1-yl)prop-2-enimidic acid	2.2108	2.0709	0.9463	9.51E-06
Prochlorperazine sulfone	1.896	1.8757	1.0402	1.10E-05
PE(16:0/0:0)	1.9226	1.7837	0.9611	1.22E-05
LysoPE(18:0/0:0)	1.7468	1.6299	0.97	1.30E-05
Alanylglycine	2.1534	2.1411	1.0491	1.55E-05
Gamma-Glutamylasparagine	2.5577	2.4052	0.909	1.75E-05
6-[(4-carboxy-2,6-dihydroxycyclohex-3-en-1-yl)oxy]-3,4,5-trihydroxyoxane-2-carboxylic acid	1.5632	1.5217	1.0244	3.01E-05
Hydroxyphenyllactic acid	1.9952	1.8588	0.954	3.97E-05
3-Methyl-L-histidine	1.3054	1.3127	1.0172	4.31E-05
6-(hydroxymethyl)-7-methoxy-2H-chromen-2-one	2.7617	2.7441	0.9109	4.61E-05
(2S)-2-hydrazinyl-3-(4-hydroxy-3-methoxyphenyl)-2-methylpropanoic acid	1.4801	1.4682	1.0205	5.31E-05
M-Coumaric acid	1.7973	1.6564	0.9489	0.0001043
2-amino-4-({1-[(carboxymethyl)-C-hydroxycarbonimidoyl]-2-[(3-oxo-1-phenylpropyl)sulfanyl]ethyl}-C-hydroxycarbonimidoyl)butanoic acid	1.4103	1.3875	0.9803	0.0001071
Deoxyguanosine	1.7701	1.7226	1.0355	0.0001411
1-Methylhistidine	2.1355	2.1183	0.9356	0.0001553
1,2,10-Trihydroxydihydro-trans-linalyl oxide 7-O-beta-D-glucopyranoside	2.5802	2.5441	0.9275	0.000156
2-O-Methylcytosine	1.3364	1.3195	0.9799	0.000158
Histidylproline diketopiperazine	2.2439	2.148	1.0615	0.000161
Austalide I	2.1607	2.1161	0.9508	0.0001754
LysoPC(16:1(9Z))	1.9086	1.8492	0.9683	0.0001836
3-Furoic acid	2.0919	2.0823	1.0885	0.0001881
1-(5-Hydroxy-2-pyrimidinyl)piperazine	1.6543	1.639	1.028	0.0001884
PE(20:4/0:0)	2.703	2.6152	0.8867	0.0001955
Hydroxyisovaleroyl carnitine	1.5342	1.4992	0.9714	0.0001984
L-Carnitine	1.3207	1.2972	1.0189	0.0001992
Prosopinine	1.8289	1.7791	0.9599	0.0002456
Dopamine glucuronide	1.6596	1.6125	1.0335	0.000248
9,12-dioxo-dodecanoic acid	1.1555	1.1423	0.9847	0.0002745
Imidazoleacetic acid	1.4808	1.4427	1.0409	0.000279
N-Acetyl-L-alanine	1.0092	0.9543	0.9857	0.000298
Indolelactic acid	1.712	1.6063	0.9615	0.0003069
4-hydroxy-crotonic acid	1.7069	1.6332	1.0363	0.0003276
Acetylisoniazid	1.2595	1.2589	1.018	0.0003744
N,N-Diacetyl-O-methylhydroxylamine	1.7799	1.7376	0.9657	0.0004072
Sonchifolin	1.8949	1.9056	0.9524	0.0004466
Pseudoecgonine	1.6837	1.5686	0.9592	0.0004505

Gly Phe	1.349	1.3407	1.0243	0.0004872
D-Maltose	1.3969	1.4289	1.0249	0.000524
Oxoadipic acid	2.0266	1.949	1.0693	0.0005355
D-Galactose	1.3264	1.2857	1.0204	0.0005605
Ozagrel	1.2637	1.2396	0.9801	0.0005632
(+/-)-17,18-DiHETE	1.6133	1.5402	0.9609	0.0006142
Citramalic acid	2.5169	2.3464	1.1175	0.0006372
Acetyl-L-tyrosine	1.0989	1.0572	1.0191	0.0006529
Hydroxyprolyl-Lysine	1.1431	1.138	1.0159	0.0008886
1-(11Z-eicosenoyl)-glycero-3-phosphate	1.4517	1.3457	0.9717	0.0008953
Chitotriose	1.6015	1.5743	0.9708	0.00099
2-amino-4-({2-[(2-benzyl-3-oxopropyl)sulfanyl]-1-[(carboxymethyl)-C-hydroxycarbonimidoyl]ethyl}-C-hydroxycarbonimidoyl)butanoic acid	1.6542	1.6189	0.9677	0.001118
5-(hydroxymethyl)-2-Furancarboxylic acid	1.2328	1.1732	1.0175	0.001371
2-Hydroxyadipic acid	1.0232	0.99	1.0096	0.001509
Norvaline	1.0618	1.0725	1.016	0.00158
Glyceraldehyde	1.3827	1.334	1.0265	0.001583
5-Methyldeoxycytidine	1.1458	1.1044	0.9849	0.001662
Alpha-Lactose	1.187	1.2259	1.0146	0.002116
Serylleucine	1.2118	1.1738	0.9844	0.002399
Glutamate, gamma-methyl ester	1.246	1.2017	1.0176	0.002627
Fluticasone 17beta-carboxylic acid	1.3557	1.3502	0.9692	0.002919
2-(3-Carboxy-3-(methylammonio)propyl)-L-histidine	1.1968	1.1959	1.0191	0.003296
D-Glucose	1.2251	1.185	1.0225	0.003509
Prolyl-Glutamine	1.8731	1.8539	0.9467	0.003715
Dinorpromazine	1.1275	1.0726	0.9827	0.003722
Benazeprilat	1.0425	0.9406	0.9859	0.003729
3,4-dichloro-tridecanoic acid	2.53	2.3799	1.0887	0.003762
B-D-Glucopyranosiduronic acid	1.5337	1.4788	1.0294	0.003813
2-Ethyl-2-Hydroxybutyric acid	1.163	1.097	0.983	0.004129
N2,N2-Dimethylguanosine	1.2021	1.1501	0.9838	0.004496
7-Methylguanine	1.1208	1.0849	0.9853	0.004649
5-Methylcytidine	1.0223	0.9889	0.9854	0.005223
Xanthosine	1.0944	1.0348	0.9816	0.005549
(E)-2-Methyl-2-buten-1-ol O-beta-D-Glucopyranoside	1.0079	0.986	1.0123	0.005593
20-Hydroxy-E4-neuroprostane	1.5457	1.4694	0.9648	0.005622
Valyl-Asparagine	1.1206	1.0836	0.9816	0.006294
Gly Leu Trp	1.2694	1.2847	1.0204	0.006734
Cucurbitine	1.1248	1.1726	1.0164	0.007457
Chlorogenoquinone	1.272	1.2404	0.9802	0.008151
Val Val	1.5568	1.5138	0.9643	0.00942
3,6-dihydroxy-4,5-diphenyl-2,3,4,5-tetrahydropyridin-2-one	1.1628	1.1235	1.0211	0.01012
N6-Acetyl-L-lysine	1.0094	1.0234	1.012	0.01031

Tyrosyl-Hydroxyproline	1.907	1.9016	0.9501	0.0109
Nitecapone	1.4327	1.3624	0.9661	0.01145
MG(13:0/0:0/0:0)	1.0012	0.9169	1.0119	0.01245
Asp His Asp Glu	1.3791	1.3567	0.9791	0.01263
Lactose 6-phosphate	1.2201	1.2011	0.979	0.01394
3,4,5-trihydroxy-6-({7-oxo-7H-furo[3,2-g]chromen-4-yl}oxy)oxane-2-carboxylic acid	1.1267	1.0542	0.9787	0.01519
Gamma-Glutamylglutamic acid	1.0814	1.0246	0.9817	0.01551
Trimethylsilyl nonanoic acid	2.1583	2.1453	1.1297	0.01625
PC(16:0/0:0)	1.1525	1.0668	0.9869	0.01643
[5-(7-hydroxy-4-oxo-4H-chromen-2-yl)-2-methoxyphenyl]oxidanesulfonic acid	2.1597	2.0587	1.0762	0.01856
6-[4-(2-carboxyeth-1-en-1-yl)-5-hydroxy-2-methoxyphenoxy]-3,4,5-trihydroxyoxane-2-carboxylic	2.1079	1.8657	1.0796	0.01908
5-Phenyl-1,3-oxazinane-2,4-dione	1.3802	1.2184	1.0252	0.01912
Uridine	1.2098	1.1671	0.9788	0.01938
(3-Arylcarbonyl)-alanine	1.054	1.0386	0.9798	0.01977
(all-E)-1,8,10-Heptadecatriene-4,6-diyne-3,12-diol	1.2572	1.2481	0.976	0.02002
Lysyl-Methionine	1.7787	1.645	0.9531	0.02049
Valyl-Hydroxyproline	1.2897	1.2935	0.975	0.02102
Trans-3,4-Dihydro-3,4-dihydroxy-7,12-dimethylbenz[a]anthracene	1.2026	1.1695	1.0306	0.02129
9H-Carbazole-3-carboxaldehyde	1.1186	1.067	1.0213	0.02153
{[3-(6-hydroxy-7-methoxy-2H-1,3-benzodioxol-5-yl)prop-2-en-1-yl]oxy}sulfonic acid	1.0485	0.9815	1.0179	0.02209
6-{4-[(1E)-3-{3-[6-carboxy-5-(2,4-dihydroxyphenyl)-3-methylcyclohex-2-en-1-yl]-2,4-dihydroxyphenyl}-3-oxoprop-1-en-1-yl]-3-hydroxyphenoxy}-3,4,5-trihydroxyoxane-2-carboxylic	1.9637	1.9563	0.955	0.02221
3,4,5-trihydroxy-6-{[2-hydroxy-3-(4-hydroxy-3-methoxyphenyl)propanoyl]oxy}oxane-2-carboxylic ac	2.5941	2.4181	1.1191	0.02226
Deoxypyridinoline	1.8798	1.9201	1.0667	0.02258
Chondroitin sulfate	1.3532	1.3091	0.969	0.02348
Tetrahydropentoxyline	1.0413	0.9909	1.0133	0.02466
Sepiapterin	1.2781	1.2715	0.9766	0.0251
Glycinexylidide	1.2116	1.1345	0.9725	0.02583
Deoxyuridine	2.1376	1.9679	1.0838	0.02871
3,4,5-trihydroxy-6-{[(2E)-2-methyl-3-phenylprop-2-en-1-yl]oxy}oxane-2-carboxylic acid	1.3195	1.2307	0.9737	0.02924
Glutamyltryptophan	1.4111	1.4124	0.9636	0.03049
3,4,5-trihydroxy-6-[(1-oxo-1H-isochromen-3-yl)methoxy]oxane-2-carboxylic acid	1.6896	1.5549	1.0469	0.03254
N-[(4-Hydroxy-3-methoxyphenyl)methyl]octanamide	1.6752	1.7252	1.0593	0.03312
3-hydroxybenzoic acid-3-O-sulphate	1.0032	1.0348	0.9809	0.03384
Gly Asp Trp Trp	1.4784	1.3692	0.9758	0.03425
Histidinal	1.5103	1.4599	0.9595	0.0353
PC(22:5/0:0)	1.0874	1.0894	0.982	0.03569
Blumealactone A	2.4058	2.3114	0.8872	0.03793
Geranyl-PP	1.0879	1.0575	1.0191	0.03866
4-PIOL	1.1869	1.1677	0.973	0.04344
Asp Glu His Asp	1.1605	1.1541	0.9817	0.0454
Xanthine	1.2123	1.2172	0.9758	0.04636
