# Supplementary material for: Adipocyte-expressed SIRT3 manipulates carnitine pool to orchestrate metabolic reprogramming and polarization of macrophages
Source: Cell Death Dis. 2025 May 15;16(1):381. doi: 10.1038/s41419-025-07699-6 (PMC12078679; doi:10.1038/s41419-025-07699-6)
Supplement: Supplementary file 3 — original Western blots for reviewers [file 41419_2025_7699_MOESM3_ESM.pdf]

Uncropped blots for Figure 1

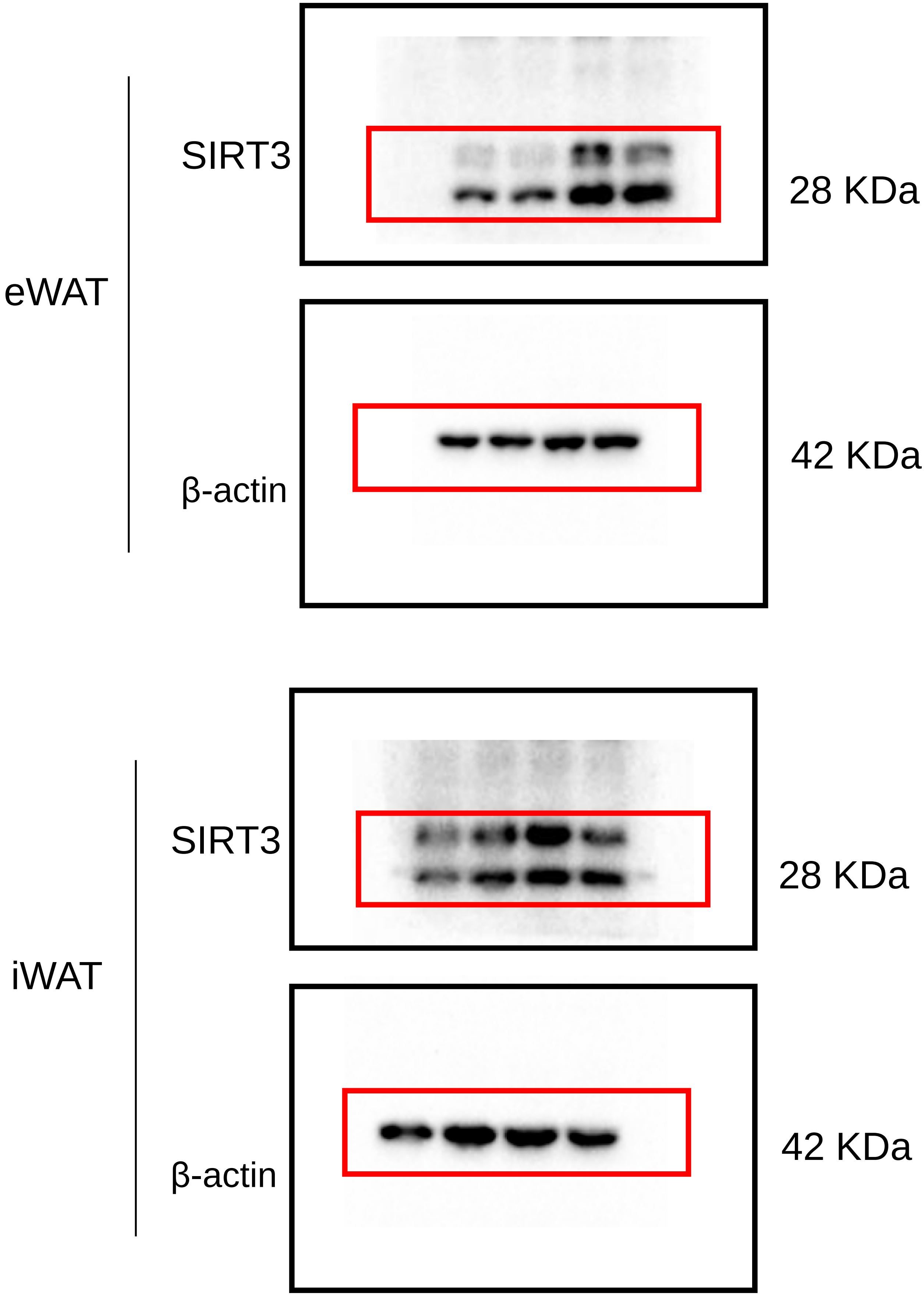

Uncropped blots for Figure 1

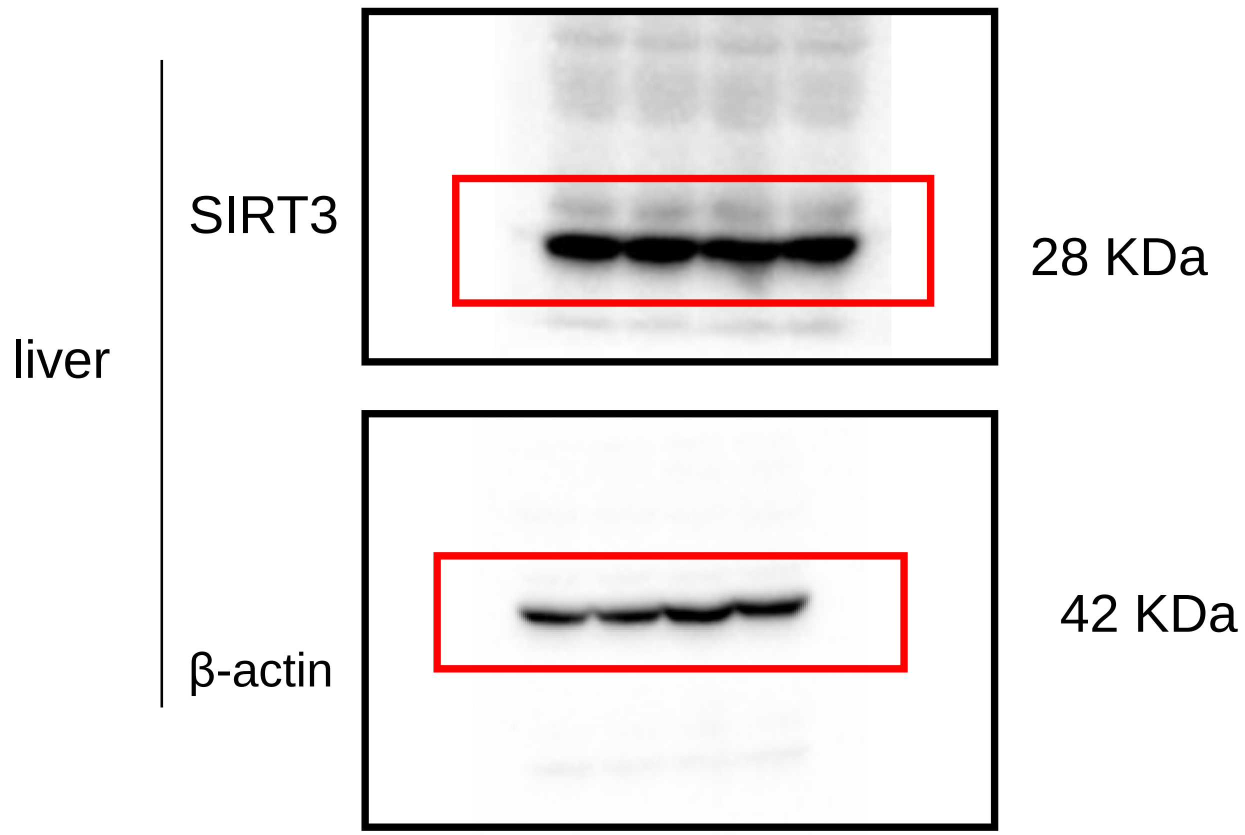

Uncropped blots for Figure 9A

Ac-lysine

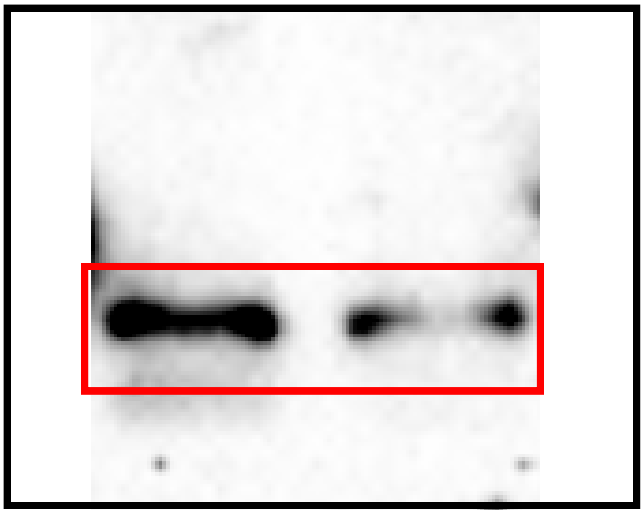

71 KDa

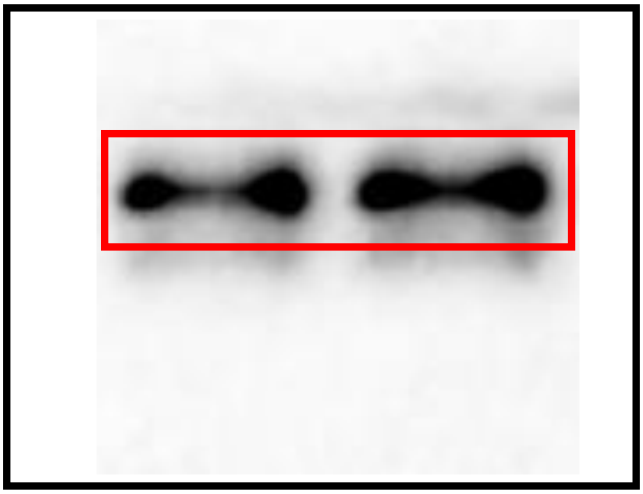

71 KDa

CPT2

Uncropped blots for Figure 9M

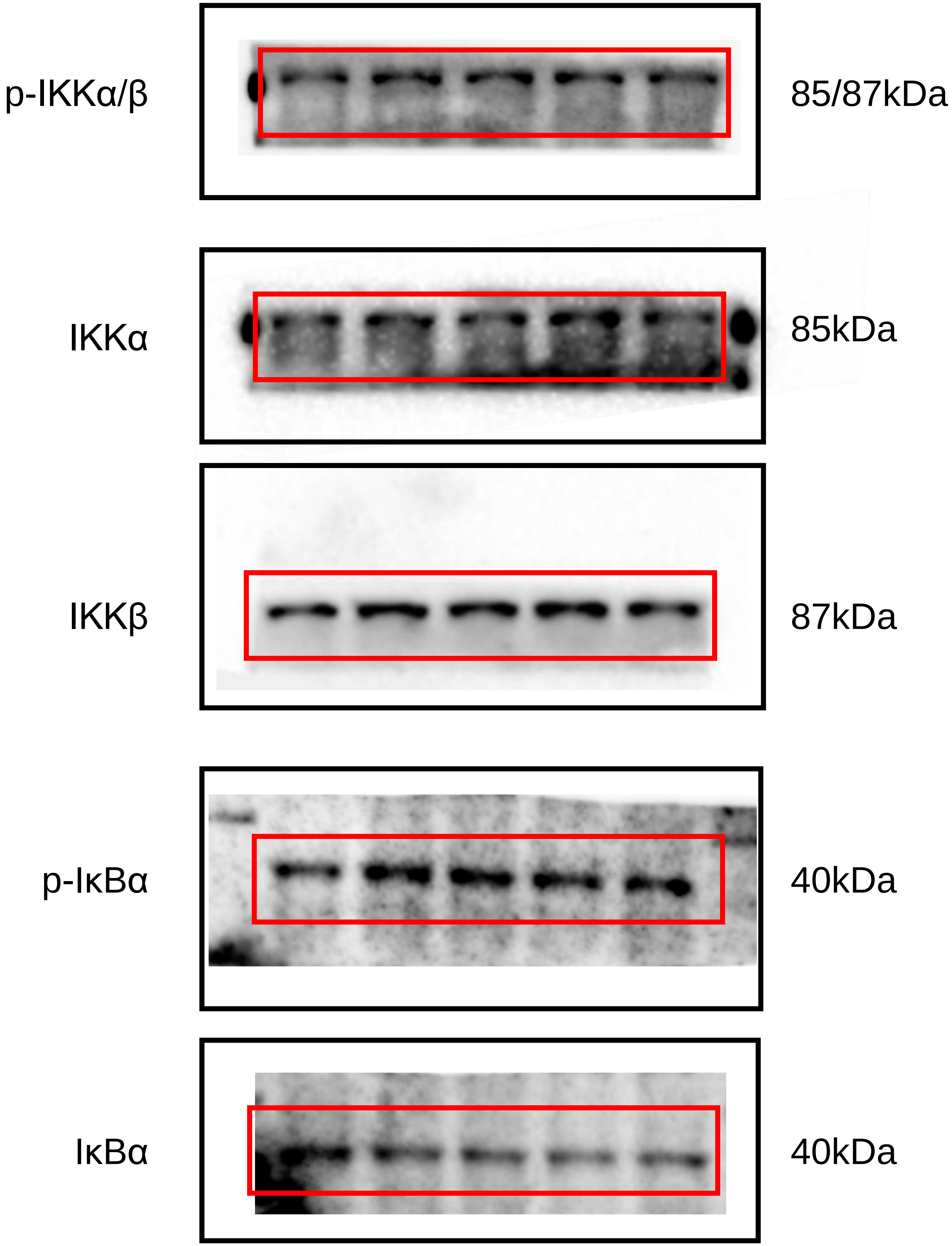

Uncropped blots for Figure 9M

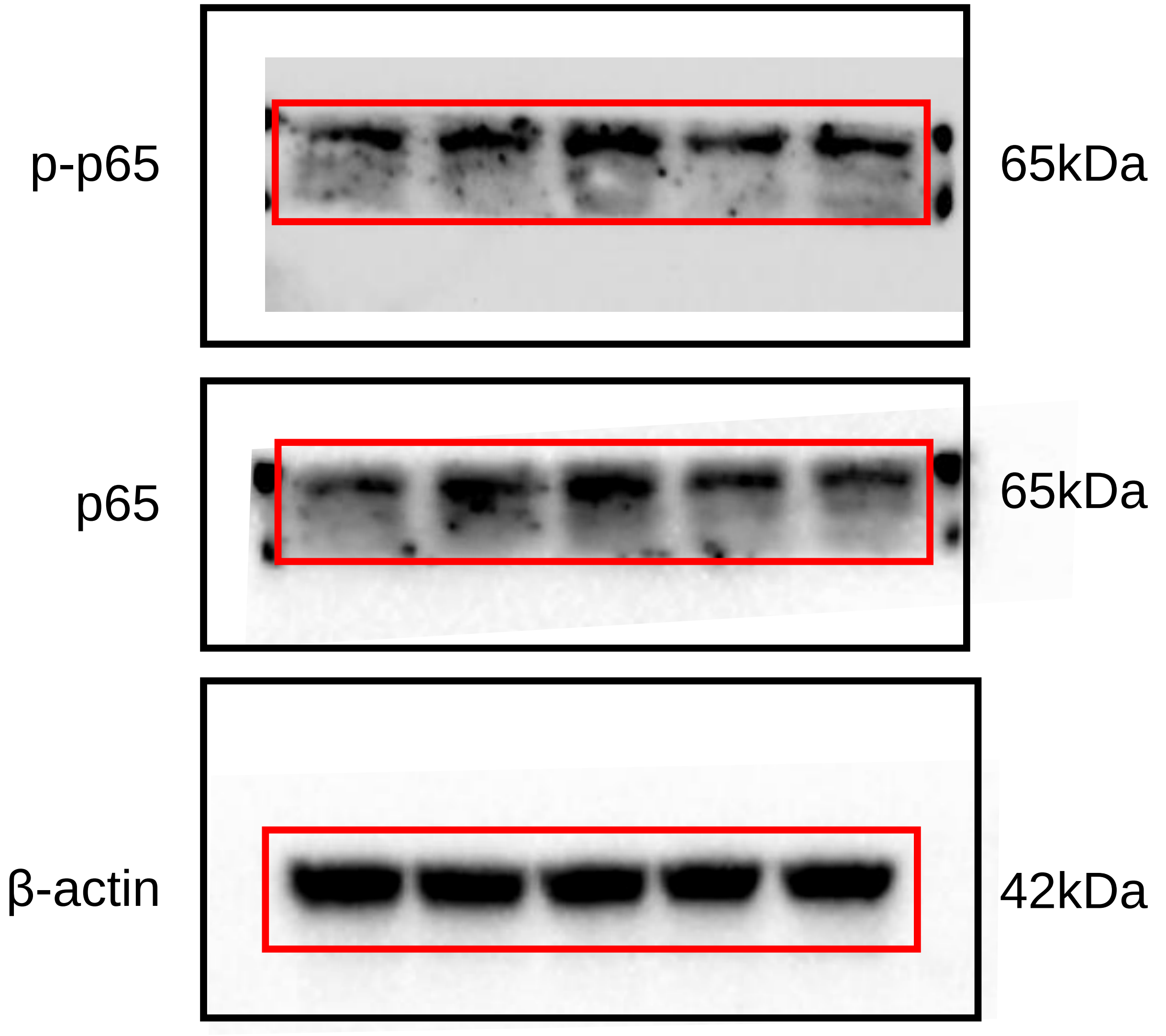

Uncropped blots for Figure S2A

SIRT3

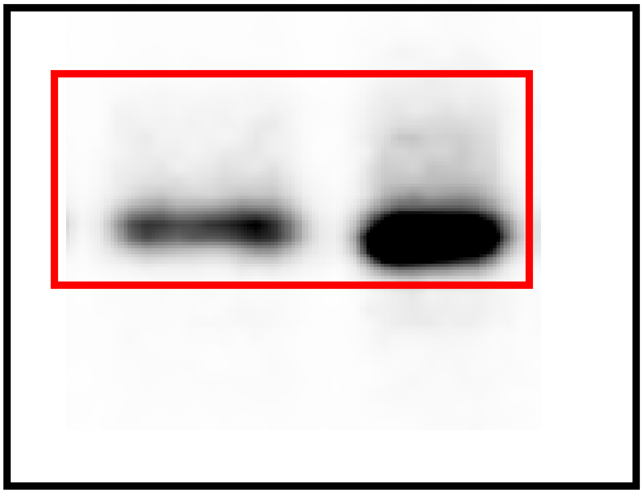

28 KDa

α-tubulin

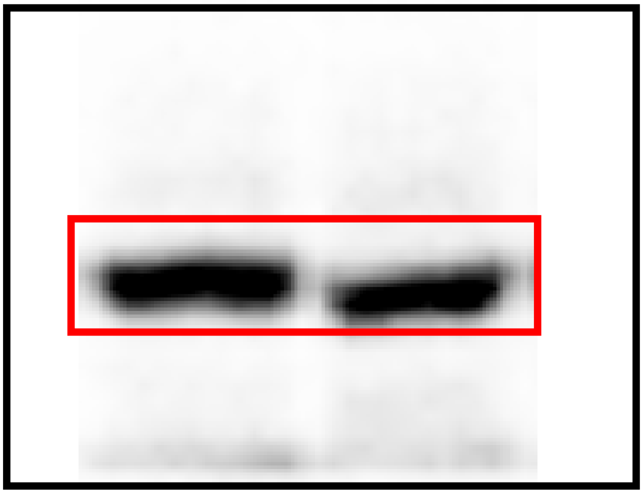

55 KDa

Uncropped blots for Figure S2B

SIRT3

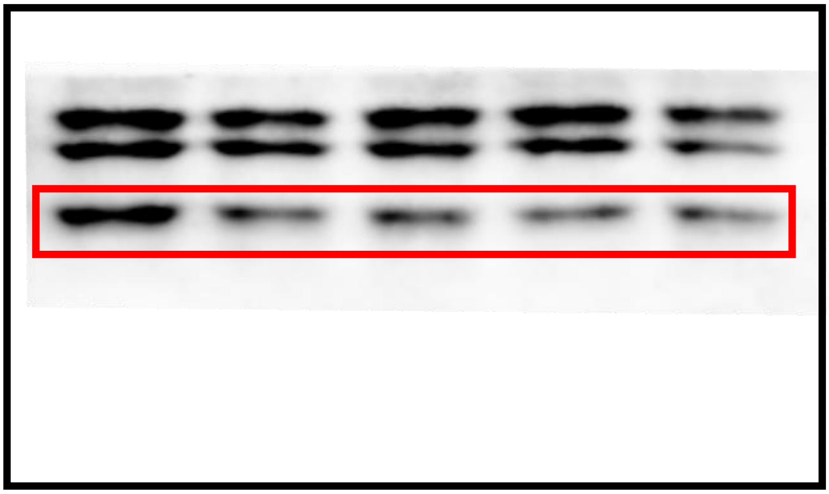

28 KDa

α-tubulin

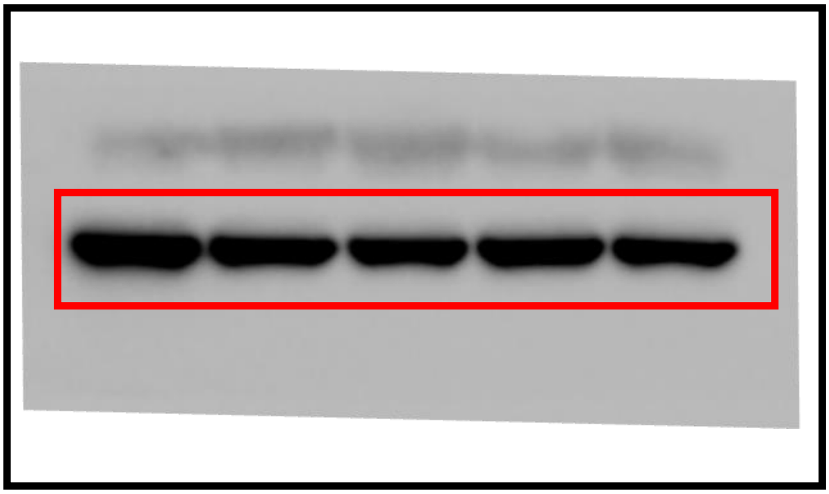

55 KDa

Uncropped blots for Figure S4

CPT2

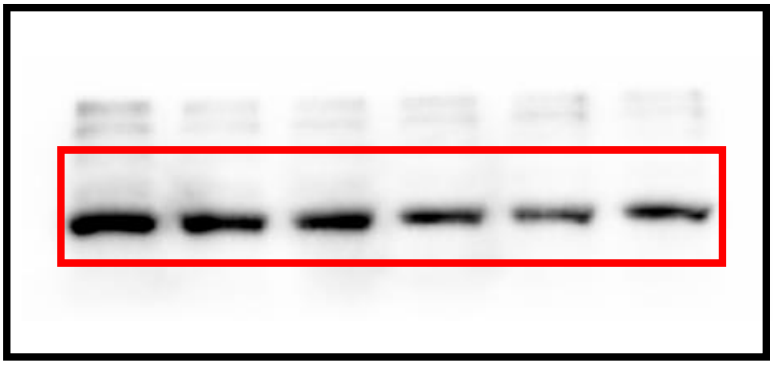

71 KDa

β-actin

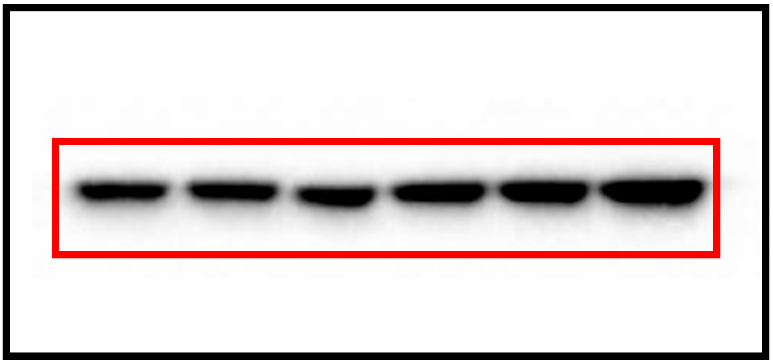

42 KDa
